# Global synonymous mutagenesis identifies cis-acting RNA elements that regulate HIV-1 splicing and replication

**DOI:** 10.1371/journal.ppat.1006824

**Published:** 2018-01-29

**Authors:** Matthew A. Takata, Steven J. Soll, Ann Emery, Daniel Blanco-Melo, Ronald Swanstrom, Paul D. Bieniasz

**Affiliations:** 1 Laboratory of Retrovirology, The Rockefeller University, New York, New York, United States of America; 2 Howard Hughes Medical Institute, The Rockefeller University, New York, New York, United States of America; 3 Curriculum in Genetics and Molecular Biology, University of North Carolina at Chapel Hill, Chapel Hill, North Carolina, United States of America; 4 Lineberger Comprehensive Cancer Center, University of North Carolina at Chapel Hill, Chapel Hill, North Carolina, United States of America; 5 Department of Microbiology and Immunology, University of North Carolina at Chapel Hill, Chapel Hill, North Carolina, United States of America; 6 Department of Biochemistry and Biophysics, University of North Carolina at Chapel Hill, Chapel Hill, North Carolina, United States of America; Fred Hutchinson Cancer Research Center, UNITED STATES

## Abstract

The ~9.5 kilobase HIV-1 genome contains RNA sequences and structures that control many aspects of viral replication, including transcription, splicing, nuclear export, translation, packaging and reverse transcription. Nonetheless, chemical probing and other approaches suggest that the HIV-1 genome may contain many more RNA secondary structures of unknown importance and function. To determine whether there are additional, undiscovered cis-acting RNA elements in the HIV-1 genome that are important for viral replication, we undertook a global silent mutagenesis experiment. Sixteen mutant proviruses containing clusters of ~50 to ~200 synonymous mutations covering nearly the entire HIV-1 protein coding sequence were designed and synthesized. Analyses of these mutant viruses resulted in their division into three phenotypic groups. Group 1 mutants exhibited near wild-type replication, Group 2 mutants exhibited replication defects accompanied by perturbed RNA splicing, and Group 3 mutants had replication defects in the absence of obvious splicing perturbation. The three phenotypes were caused by mutations that exhibited a clear regional bias in their distribution along the viral genome, and those that caused replication defects all caused reductions in the level of unspliced RNA. We characterized in detail the underlying defects for Group 2 mutants. Second-site revertants that enabled viral replication could be derived for Group 2 mutants, and generally contained point mutations that reduced the utilization of proximal splice sites. Mapping of the changes responsible for splicing perturbations in Group 2 viruses revealed the presence of several RNA sequences that apparently suppressed the use of cryptic or canonical splice sites. Some sequences that affected splicing were diffusely distributed, while others could be mapped to discrete elements, proximal or distal to the affected splice site(s). Overall, our data indicate complex negative regulation of HIV-1 splicing by RNA elements in various regions of the HIV-1 genome that enable balanced splicing and viral replication.

## Author summary

In addition to encoding viral proteins, the HIV-1 genome contains sequence elements that act at the level of RNA to enable replication. We undertook an experiment to discover new RNA elements that act in this way by altering nearly the entire coding sequence of the viral genome so as to change the RNA sequence without changing protein sequences. This experiment uncovered two classes of defective mutants. One class had profound defects in RNA splicing, the other had no obvious defects in splicing. Through an analysis of the splicing-defective mutants, we found several previously RNA sequences in the viral genome that affected splicing, enabling a nearly complete catalogue of signals that regulate HIV-1 alternative splicing in infected cells to be derived. Because these newly described sequences lack sequence motifs that are known to bind to canonical splicing-regulatory proteins, they may function through novel mechanisms.

## Introduction

The HIV-1 genome contains a variety of RNA elements that have important cis-acting functions [1, 2]. Some of these RNA sequences are multi-functional in that they lie in open reading frames and therefore encode proteins as well as performing functions as RNA that are critical during viral replication. Known cis-acting RNA elements in the HIV-1 genome that lie within protein-coding sequences include splice donors, acceptors, and branch points [3], splicing regulatory elements that enhance or inhibit the use of proximal splice sites [4], the Rev-responsive element [5, 6], the central polypurine tract and termination sequence [7], the Gag-Pro-Pol ribosomal frameshift regulatory element [8] and components of the viral genome packaging signal [9, 10]. It is not known whether the aforementioned represent a complete catalogue of cis-acting RNA elements, or whether additional RNA-based functionality exists in the HIV-1 genome. That additional RNA sequences function in cis may exist in the HIV-1 genome is suggested by studies employing chemical probing approaches. For example, SHAPE experiments indicate that individual nucleotides in HIV-1 RNA have widely divergent tendencies to be base-paired [11–13]. These findings, along with phylogeny-based approaches, strongly suggest that secondary structures form in HIV-1 RNA that are conserved between strains, and might therefore serve a function in HIV-1 replication [11, 13]. One example of a recently suggested function for HIV-1 RNA secondary structure is the regulation of translational rate, whereby translation is periodically slowed to enable folding of one protein domain of the multidomain HIV-1 Gag protein before synthesis of the next [11]. However, no evidence for such a phenomenon was found in an analysis of SIVmac [14].

Despite the suggestion that novel RNA secondary structures may be important for HIV-1 replication, targeted mutagenesis of putatively important individual stem loops has not generally yielded evidence that is strongly supportive of a role for these potential structures in HIV-1 replication [15]. Conversely, some studies in which portions of the HIV-1 genome were synonymously mutated have suggested a role for RNA (as opposed to protein) sequence or structure in HIV-1 replication [16, 17]. However, the precise nature of defects induced by synonymous mutations are unclear, and possibly pleiotropic.

**Fig 1:**
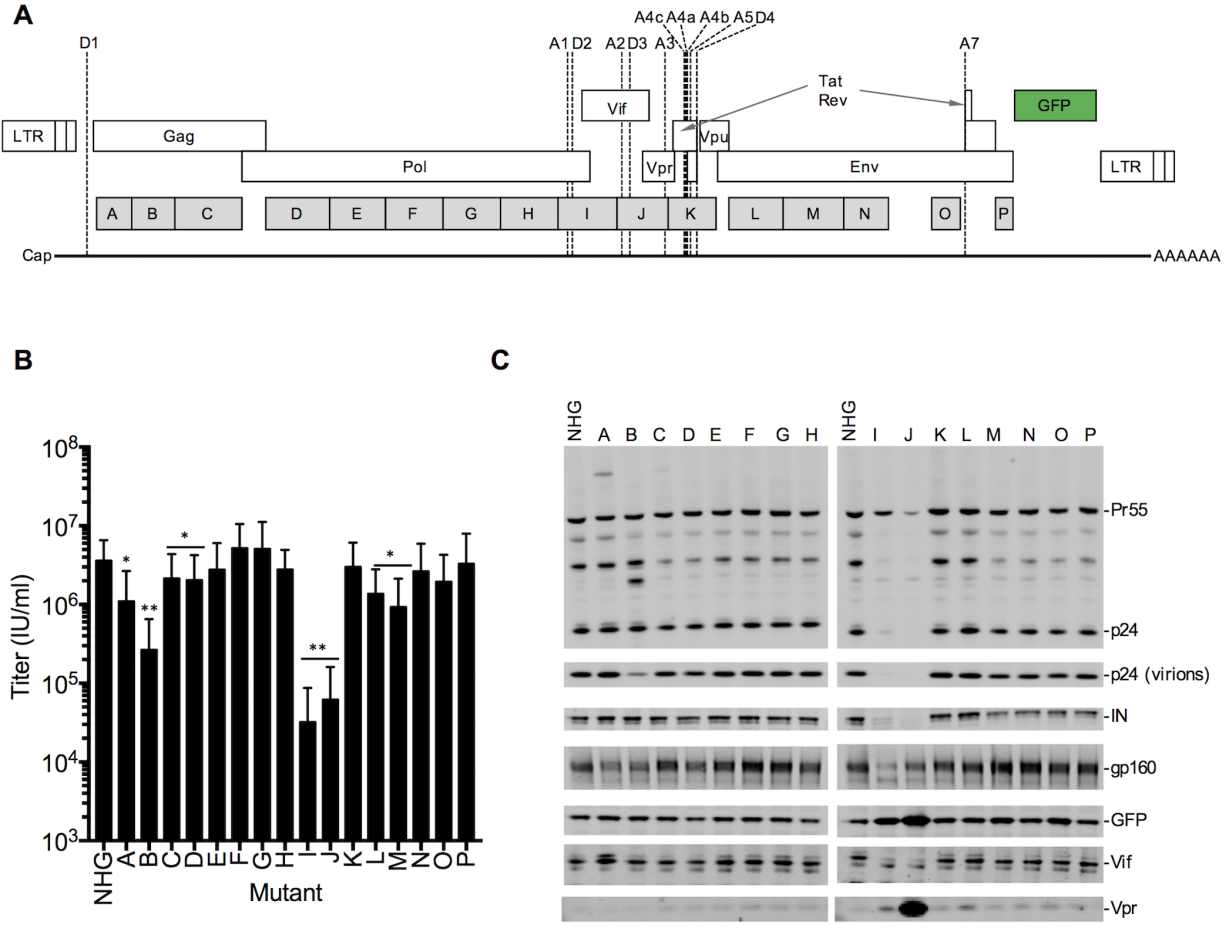
Design and analysis of panel of synonymously mutated HIV-1 viruses. (A) Schematic of HIV-1 proviral DNA, indicating open reading frames, splice sites, and blocks of nucleotides that were synonymously mutated in the 16 proviral plasmids (A-P). (B) Single-cycle infectious titers (measured using MT4 cells) 48h following transfection of 293T cells with each of the WT(HIV-1_NHG_) and mutant (A-P) proviral plasmids. Valuses are given as mean±sd. (n = 3) *p<0.05, **p<0.005 by students t-test, calculated with relative values normalized to WT values in each experiment. (C) Western blot analysis of protein levels in transfected cells (and virion particles where indicated) at 48h after transfection of 293T cells with each of the WT(HIV-1_NHG_) and mutant (A-P) proviral plasmids.

RNA sequence and structure must play a key role in a particular aspect of HIV-1 replication, namely alternative splicing. Indeed, RNA secondary structure surrounding the major 5’ splice donor affects splicing [18, 19] and SHAPE analysis revealed a novel stem loop that influences splicing [14, 20]. Nevertheless, the regulatory mechanisms controlling alternative splicing are incompletely understood [4, 21]. HIV-1 employs four salient splice donors (D1, D2, D3, D4) and eight acceptors (A1, A2, A3, A4a,b,c, A5 and A7, S1 Table) to generate a large number of mRNAs that enable expression of nine viral open reading frames in a temporally biphasic manner [22]. Additional, ‘cryptic’ splice sites may be used at very low frequency, and are not required for expression of the viral ORFs (S1 Table). HIV-1 splicing is, by necessity, inefficient as a substantial fraction of the viral transcripts must remain unspliced so as to provide viral genomes and Gag-Pol mRNA [21, 23]. Splice-site utilization is regulated by deviation from consensus splicing signals as well as regulatory elements consisting of exonic and intronic splicing silencer (ESS and ISS) elements, and exonic splicing enhancer (ESE) elements [4, 21, 23–32] (S1 Table). All HIV-1 splicing involves D1, while splice acceptor sites 5’ to each initiation codon are used to generate mRNAs encoding the HIV-1 proteins Vif (A1), Vpr (A2), Tat (A3) Rev (A4a,b,c), Vpu/Env (A4a,b,c and A5) and Nef (A5 and A7) (Fig 1) [3, 4]. In addition, some HIV-1 mRNAs include short noncoding exons (SX1 (A1-D2)) and (SX2 (A2-D3)) 5’ to the expressed open reading frame. The relative frequencies that the various splice sites are used, which can be measured using next-generation sequencing approaches [20, 33], likely contributes to ensuring that the optimal levels of viral proteins are synthesized for viral replication.

To identify and catalog regions of the HIV-1 genome that contain critical but unidentified cis-acting RNA elements that impact splicing and other functions, we employed a global synonymous mutagenesis strategy. Sixteen mutant proviruses, each containing blocks of synonymous mutations covering nearly the entire HIV-1 protein coding sequence, were examined. Despite encoding identical proteins, and despite the fact that all known cis-acting sequences were maintained in an intact form, some viruses were completely incapable of a spreading infection. Others mutants displayed apparently normal fitness. Some defective mutants, termed Group 3 mutants, displayed near normal splicing but were defective in spreading replication assays. In these cases, second site revertants could not be recovered, and we have recently demonstrated that these defects are associated with dinucleotide compositional changes that confer sensitivity to zinc finger antiviral protein (ZAP) [34]. In this study, we focused on Group 2 mutants that displayed aberrant splicing. We found that replication competence could be recovered by second-site revertant point mutations that were often, but not always, proximal to splice sites. Sequences responsible for aberrant splicing were sometimes diffusely distributed, but sometimes could be mapped to discrete elements of 20–120 nucleotides and suggest the existence of novel forms of splicing-suppressive RNA signals. Overall, this study demonstrates that uncharacterized elements in the HIV-1 genome determine the fate and splicing of HIV-1 RNA and thus the ability of HIV-1 to replicate.

## Results

### Design and synthesis of synonymously mutated HIV-1 strains

To determine whether there are important, undiscovered cis-acting elements in the HIV-1 genome, a mutant HIV-1 sequence was designed that contained a maximum number of synonymous mutations in the open reading frames while leaving known RNA elements that are important for virus replication intact (S1 Table). Mutations were designed so as to maximize the probability of disrupting base pairing in which a given nucleotide might participate. Thus, where multiple synonymous mutation possibilities were available, transversion mutations (purine to pyrimidine or vice versa) were preferred over transition mutations. To avoid the creation of new splice acceptors and donors, no new AG or GU dinucleotides were introduced. Moreover, sequences encoding overlapping open reading frames were not altered, and all known RNA elements that control HIV-1 splicing, gene expression and reverse transcription (S1 Table) remained intact in the mutant viral genome.

This designed HIV-1 sequence contained 1,976 synonymous mutations (S2 Table, S1 Data, S2 Data) and was divided into 150–500 nucleotide blocks, that were synthesized separately. Each synthetic mutated fragment was introduced into a replication competent HIV-1 proviral plasmid (HIV-1_NHG_) [35] that carried GFP in place of the nonessential gene Nef (S2 Table, S1 Data, S2 Data.). Thus, sixteen different mutated proviral plasmids, designated A through P, with each carrying a mean of ~125 synonymous mutations were generated (Fig 1A).

### Protein expression and replication properties of synonymously mutated HIV-1 mutants

Each of the synyonymously mutated HIV-1_NHG_ proviral plasmids was transfected into 293T cells and the infectious virion yield was determined in a single-cycle infection assay in MT4 cells (Fig 1B). Many of the mutants yielded WT, or close to WT, levels of infectious virions in this transfection/titration assay format. However, mutants A, B, I, and J yielded between 5-fold and 1000-fold fewer infectious virions (Fig 1B). Western blot analysis of the transfected 293T cells and extracellular virions showed that mutants A and B expressed additional Gag protein species of unexpected sizes, and mutant B displayed a particle release defect, possibly a consequence of the expression of the aberrant Gag protein (Fig 1C). Mutants I and J displayed reduced Gag, Pol, Env and Vif protein, and slightly elevated GFP levels. Mutant J also grossly overexpressed the Vpr protein (Fig 1C).

**Fig 2:**
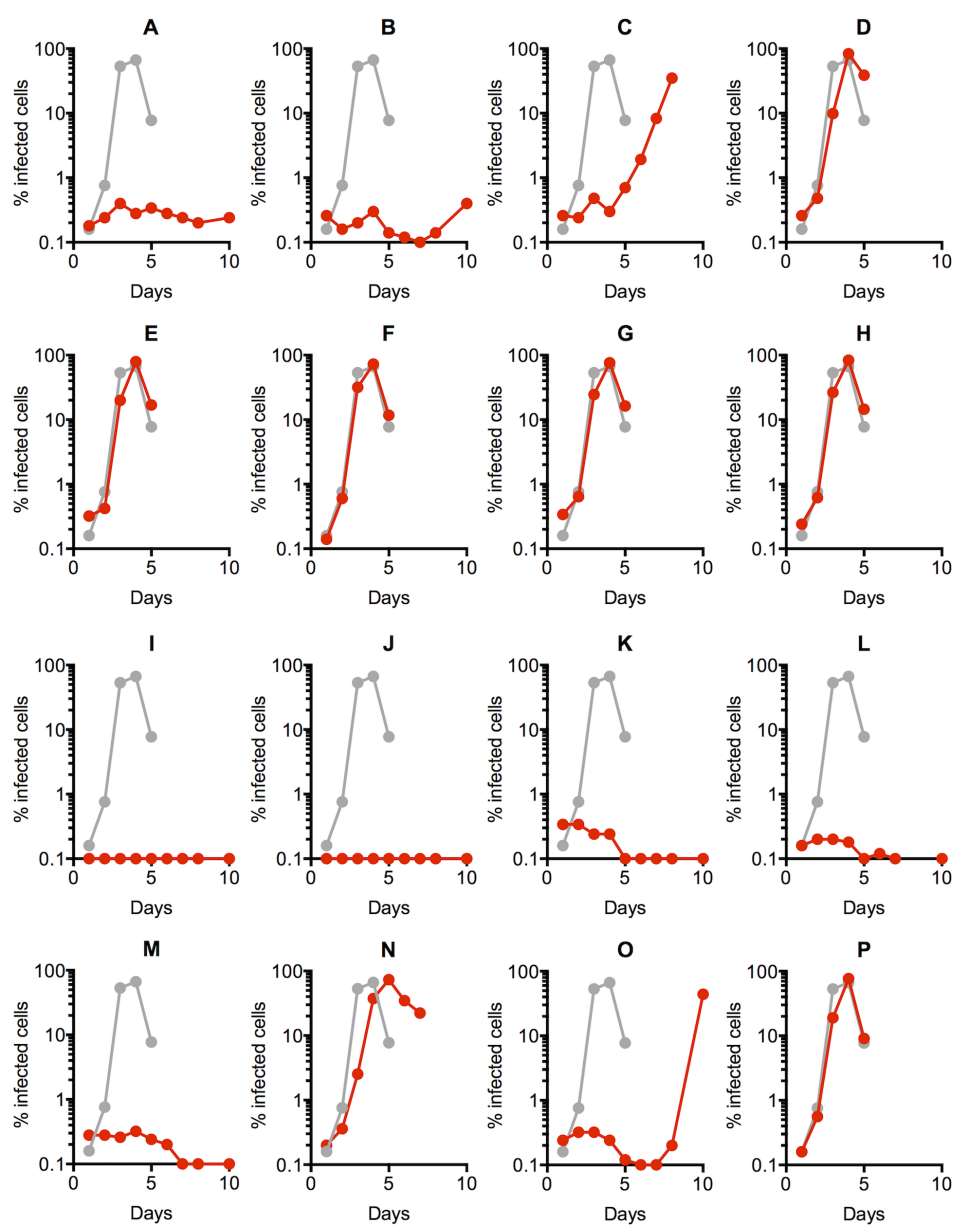
Spreading replication properties of mutant viruses. (A-P) MT4 cells were infected with the indicated virus (harvested from the supernatant of 293T cells transfected with each of the WT(HIV-1_NHG_) or mutant (A-P) proviral plasmids at an MOI of 0.002. Aliquots of infected cells were withdrawn each day, fixed in 4% PFA and the proportion of infected cells determined by FACS analysis of GFP expression. a Representative replication curve for WT(HIV-1_NHG_) is plotted in each chart as grey symbols and line, while each mutant is plotted using red symbols and lines.

We next examined whether each of the mutants could replicate in MT4 and CEM T-cell lines (Fig 2 and S1 Fig). Seven of the mutants (D, E, F, G, H, N and P) replicated with WT, or close to WT, kinetics while eight other mutants, (A, B, I, J, K, L, M, and O) were replication defective, or highly impaired, in both cell lines (Fig 2 and S1 Fig.). Mutant C was somewhat impaired in MT4 cells, but replicated with close to WT kinetics in CEM cells. Thus, an apparent discrepancy was evident in the ability of some of the mutants to generate infectious virions in 293T cells, versus their ability to generate a spreading infection in T-cell lines.

### Assays for splicing perturbations in replication-defective HIV-1 mutants

**Fig 3:**
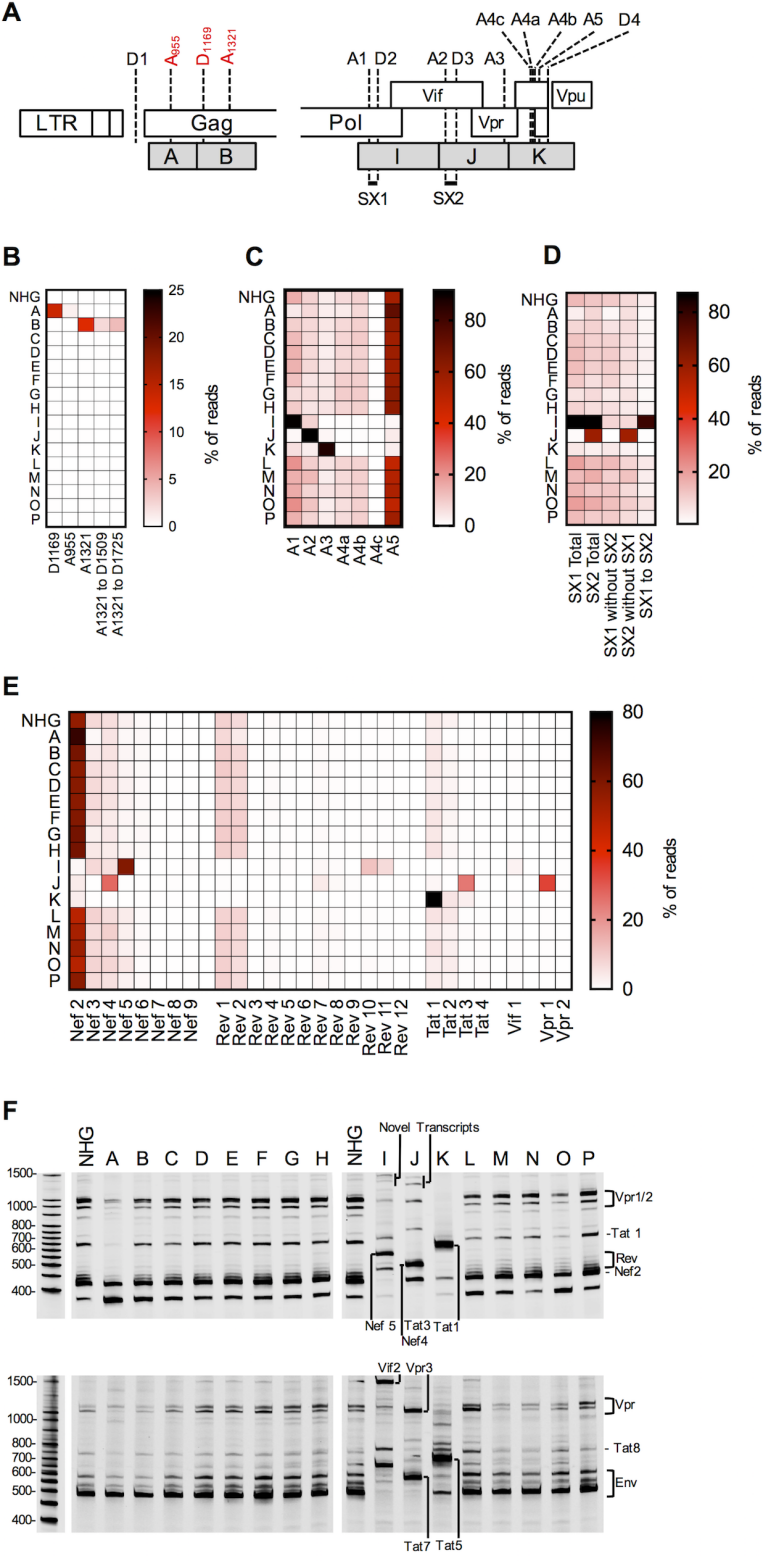
Analysis of HIV-1 splicing in WT and synonymously mutated HIV-1. (A) Schematic representation of segments of HIV-1 proviral DNA, focused on mutants exhibiting perturbed splicing. Canonical splice sites (black) and cryptic splice sites (red) are indicated, as are blocks of nucleotides that were synonymously mutated in the viruses exhibiting perturbed splicing. (B-E) Nextgen sequencing analysis of HIV-1 splicing, heatmaps indicate relative proportion of sequencing reads that indicate splicing at the sites indicated at the bottom of the heatmaps (B, C), or inclusion of the short exons (SX1 and/or SX2) indicated at the bottom of the heatmap (D). For panel (C) only direct splicing to the indicated acceptor sites is indicated in the heatmap. Alternatively, the relative abundance of the various 1.8 kb mRNA species is indicated (E). (F) Fluorescent primer PCR analysis of HIV-1 splicing. 293T cells were transfected with the indicated proviruses, RNA extracted and cDNA synthesized. A sense PCR primer situated 5’ to the major splice donor, along with an antisense primer positioned either 3’ to A7 or 3’ to D4 (labelled with IRD800) were used to amplify cDNAs derived from the 1.8 kb (top) or 4 kb (bottom) classes of spliced HIV-1 mRNAs respectively. PCR products were subjected to PAGE and a LI-COR Odyssey scanner was used to detect fluorescent signals directly from the gels.

The viral mutants were designed to avoid altering RNA sequences in the HIV-1 genome that are known to be important for replication, including those that participate in or regulate splicing (S1 Table). Nevertheless, the aberrant pattern of viral protein expression in two of the synonymously mutated viruses (I, J), and the appearance of novel Gag-related protein species in two others (A, B), suggested that HIV-1 splicing might have been perturbed in at least some of the mutant viruses (Fig 1C). Therefore, we next used two approaches to determine whether the mutations affected splicing in each of the mutant viruses. We used a recently described Primer ID-based deep sequencing approach [20] to globally quantify the relative utilization of the various splice donors and acceptors in the mutant viruses (Fig 3A–3E). We also used a fluorescent primer-based PCR-PAGE assay to more conveniently, albeit less quantitatively, track the generation of the major mRNA species in the canonical spliced 1.8 kb class of HIV-1 mRNAs (Fig 3F). These two assays yielded results that were in good agreement (see below). Of the canonical splice acceptors in the central portion of the genome (A1, A2, A3 A4a,b,c, and A5, Fig 3A), the WT HIV-1_NHG_ most frequently spliced to A5, with lower levels of splicing to A1, A2, A3, A4a,b,c (Fig 3A, 3C and 3F).

### Activation of cryptic splice sites in mutants A and B (Group 2a)

A splicing defect was observed for mutants A and B, which contained synonymous changes toward the 5’ end of the HIV-1 genome, within *gag*. For each of these mutants, the relative levels of canonical splice site utilization were only marginally perturbed, but the Primer ID-based sequencing assay revealed that cryptic splice sites near the 5’ end of the genome were activated (Fig 3B, 3C and 3D). These mutants were designated Group 2a. For mutant A, a cryptic splice acceptor at position 955 (A_955_) and a donor at position 1169 (D_1169_) were activated, neither of which was used at a measurable level in WT HIV-1_NHG_ (Fig 3A and 3B). While A_955_ was used rarely (~1% of 1.8 kb mRNAs) in mutant A, ~14% of the 1.8 kb mRNAs were spliced using D_1169_. (Fig 3B). Splicing events involving D_1169_ were selective with respect to which of the downstream acceptors were used: A3, A4, or A5, were used as acceptors for D_1169_ but A1 or A2 were not. MaxEnt scoring, which employs an *in silico* analysis tool that predicts the intrinsic splicing efficiency of splice acceptors and donor sequences [36], indicated that our mutagenesis increased the score of the cryptic acceptor A955 from -9.88 to 1.75, suggesting that it became a stronger splice acceptor (S3 Table). Thus, the minimal activation of A955 (used in 1% of spliced reads) in mutant A, could possibly be explained by a direct effect of the mutations. However, the predominant defect in mutant A was the activation of D_1169_, which lies outside the mutated region (Fig 3A) and whose MaxEnt score was not altered. Thus, activation of D_1169_ could not be due to increased intrinsic efficiency of this cryptic donor, but rather due to some other mechanism acting via RNA sequences distal to D_1169_.

For mutant B, the major perturbation was activation of a cryptic splice acceptor at position 1321 (A_1321_, Fig 3A and 3B). This splicing event, involving ~12% of mRNAs in the 1.8 kb class, appeared to enable a cascade of further alternative splicing events, 50% of which subsequently involved cryptic splice donor activation at either D_1509_ or D_1725_, outside the B mutant region (Fig 3B). However, the most obvious outcome of the aberrant splicing event was the generation of a truncated ~40 kD Gag protein (Fig 2B). This protein would be the expected translation product of an mRNA in which a D1- A_1321_ splice, and no further splicing, had occurred. Specifically, translation initiation at the second Met codon in the *gag* gene would generate a truncated ~40 kD Gag protein lacking MA, that likely accounts for the aberrant band on the cell-associated Gag western blot as well as the reduced particle yield from cells transfected with the B mutant proviral plasmid (Fig 1C).

For mutant B, the activation of A_1321_ may be due to a direct effect of the mutations increasing its MaxEnt score from -2.94 to 7.03, again suggesting it became a stronger splice acceptor (S3 Table). The other cryptic sites activated, D_1509_ and D_1725_, are both outside of the mutated region in mutant B, and their use was likely secondary to activation of A_1321_.

### Overuse of canonical splice acceptor sites in mutants I, J and K (Group 2b)

Viruses containing mutations in the central portion of the genome, specifically mutants I, J and K, exhibited a different type of splicing defect, and were designated Group 2b. Specifically, mutants I, J and K exhibited increased direct splicing to canonical splice acceptors, A1, A2 and A3 respectively, at the expense of direct splicing to downstream (3’) acceptors (Fig 3C). In the case of mutant I, the primary defect (increased use of A1) was accompanied by increased use of the proximal downstream splice donor (D2) as well as a downstream acceptor–donor pair (A2 and D3) and thus the abundant inclusion of short exons (SX) 1 and 2 (SX1 = [A1-D2] and SX2 = [A2-D3]) into spliced viral mRNAs (Fig 3D). Some elevation of the use of upstream cryptic splice sites in mutant I (D_3569_, D_3969_, D_4641_ and A_4834_) also generated low levels of novel transcripts (Fig 3F). For mutant J, overuse of A2 (which is positioned 3’ to SX1) was accompanied by overuse of proximal downstream splice donor D3 and thus overrepresentation of SX2 = [A2-D3] into spliced viral mRNAs (Fig 3D). Additionally, some utilization of cryptic splice donors (D_5052_, D_5434_, and D_5478_) generated low levels of novel transcripts (Fig 3F). For mutant K, overuse of A3 (which is positioned 3’ to SX1 and SX2) did not result in the more frequent inclusion of these short exons (Fig 3D). Overall therefore, it appeared that one consequence of the overuse of a given splice acceptor (A1 or A2), was a resultant overuse of the proximal downstream splice donor (D2 or D3), consistent with an ‘exon definition’ model of splicing control.

This overuse of canonical splice acceptors in I, J and K resulted in aberrant representation of particular viral mRNAs. Among the 1.8 kb class of mRNAs, for WT HIV-1, Nef2 was the dominant mRNA species in both splicing assays (Fig 3E and 3F). Conversely, in mutant I, Nef5 was the dominant mRNA, while in mutant J, Nef4, Tat3 and Vpr1 were overrepresented (Fig 3E and 3F). These changes were likely responsible for the overexpression of GFP (in the *nef* position) and/or Vpr in these mutants (Fig 1C). For mutant K, Tat1 mRNA was over-represented (Fig 3E and 3F), but the overall levels of protein expression were not greatly affected in transfected cells.

### Viruses with blocks of synonymous mutations with three different phenotypes that map to distinct regions of the HIV-1 genome

**Fig 4:**
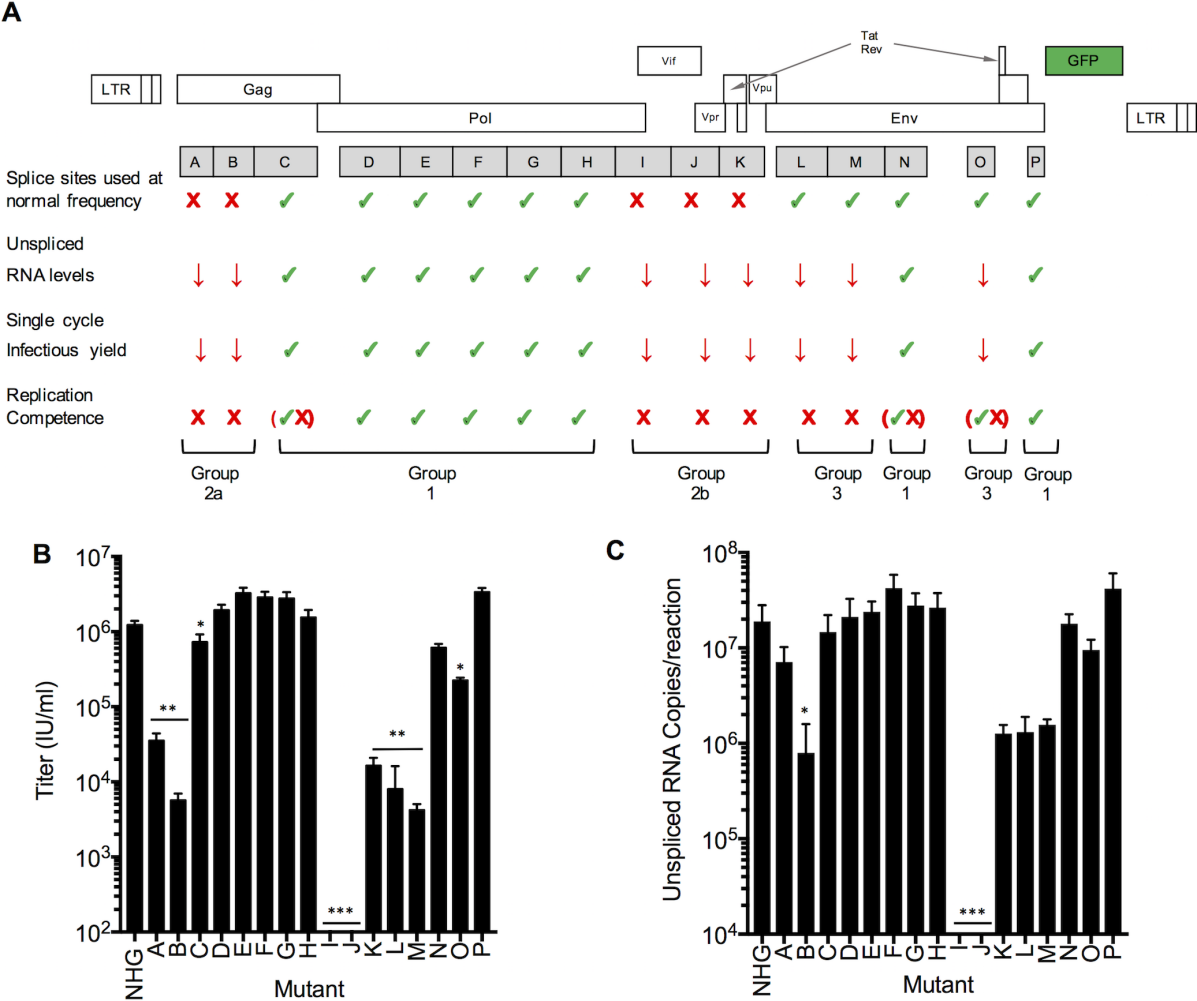
Phenotypes of synonymously mutated HIV-1 viruses. (A) Summary of the properties of HIV-1 viruses carrying blocks of nucleotides that were synonymously mutated (A-P). The frequency of splice site utilization was assessed in transfected 293T cells (Fig 3), Single-cycle replication assays were used to assess unspliced RNA levels and infectious virus yield (see panels B and C below). Replication competence was determined using spreading replication assays (Fig 2 and S1 Fig). (B) Infectious virion yield measured in the supernatant of MT4 cells, infected with each of the mutant viruses at an MOI of 1.0, and harvested 2 days post infection. Values are the mean ±sd n = 3 or n = 2 experiments, *p<0.05, **p<0.005 by students t-test calculated with relative values compared to wildtype virus (*** Values were below the limit of quantitation). (C) Levels of unspliced HIV-1 genomes in RNA extracted from MT4 cells, infected with each of the mutant viruses at an MOI of 1.0, and harvested 2 days post infection, mean ±sd n = 3 or n = 2 experiments, *p<0.05 by students t-test compared to wildtype virus. (*** Values were below the limit of quantitation).

For other replication defective mutants (L, M, and O) that we termed Group 3, the relative uses of splice sites appeared normal, despite obvious replication defects (Fig 2, Fig 3B–3E). These viruses appeared to express a normal complement of viral proteins in transfected cells (Fig 1C). Overall, therefore, analyses of viral replication and RNA splicing led to the classification of the synonymously mutated viruses into three groups (Fig 4A): Group 1 mutants exhibited near WT fitness, Group 2 mutants exhibited replication defects accompanied by perturbed RNA splicing, while Group 3 mutants had profound replication defects in the absence of obvious splicing perturbation. The three phenotypes were caused by mutations that exhibited a clear regional bias with respect to their distribution along the viral genome (Fig 4A). Specifically, Group 1 viruses carried mutations throughout the *pro* (D) and *pol* (E to H) genes or in the 3’ portion of the *env* gene (N, P), and replicated indistinguishably from HIV-1_NHG_. Conversely, Group 2 viruses with obvious splicing defects carried mutations in two distinct genomic regions: Group 2a viruses (A, B) carried mutations toward the 5’ end of the genome, within *gag*, while Group 2b viruses (I, J, K) carried mutations or in the central portions of the genome, within the accessory genes (Fig 4A). Group 3 viruses (L, M, O) that were defective but exhibited near-normal splicing carried mutations in the *env* gene (Fig 4A).

### Single-cycle replication assays suggest that reduced levels of unspliced viral RNA underlie replication defects in synonymously mutated HIV-1

We next explored the discrepancy in the abilities of certain mutant viruses to yield infectious virions after transfection of 293T cells, but were unable to spread in MT4 cells (Fig 2). We examined this discrepancy by carrying out experiments that analyzed a single cycle of replication in MT4 cells. Specifically, we infected cells with each of the mutant viruses at an M.O.I. of 1 and quantified infectious virion release (Fig 4B) as well as levels of full length unspliced viral mRNA, during the first cycle of infection (Fig 4C). These experiments revealed significant deficiencies in infectious virion yield from infected cells for mutants A, B, K, L and M, that were not evident or less evident when virions were generated after transfection of 293T cells with a plasmid containing full length viral DNA (Fig 4B, Fig 1B). Moreover, these deficiencies, and the inability of each mutant virus to support a spreading infection, correlated with reduced levels of unspliced HIV-1 RNA in infected cells (Fig 4A and 4C). These observations suggest that the underlying defect in each of the replication defective mutants is a deficit in maintaining the level of unspliced RNA. This deficiency likely result from excessive or aberrant splicing for Group 2 mutants. Conversely we have recently shown that at least one of the Group 3 viruses was sensitized to the antiviral protein, ZAP, through the cumulative effect of incidentally included CG dinucleotides during the synonymous mutagenesis [34]. It therefore appears likely that the deficit in unspliced RNA can be overcome, for some mutants, through the overexpression that results from transient viral DNA transfection in 293T cells. However, each of the mutants with reduced levels of unspliced RNA in single cycle-infected cells was profoundly defective in a spreading replication assay (Fig 4A).

### Mapping elements responsible for splicing perturbations in Group 2 viruses

To determine the sequence elements responsible for the perturbations in splicing in Group 2a and Group 2b viruses we took two approaches. First, we attempted to derive second-site revertant viruses through passage of each viral mutant in MT4 cells. Second, we applied a mapping approach, in which each block of mutations in mutants A, B, I, J and K was divided into roughly equally sized component segments (S2 Table), and the splicing properties of each secondary mutant re-examined. Through an iterative process, sometimes combining mapping and second-site revertant derivation, we could determine the nature of the defects in each Group 2 mutant and map the responsible cis-acting sequences.

### Native HIV-1 RNA sequence confers suppression of cryptic splice sites that are activated in Group 2a mutant viruses

**Fig 5:**
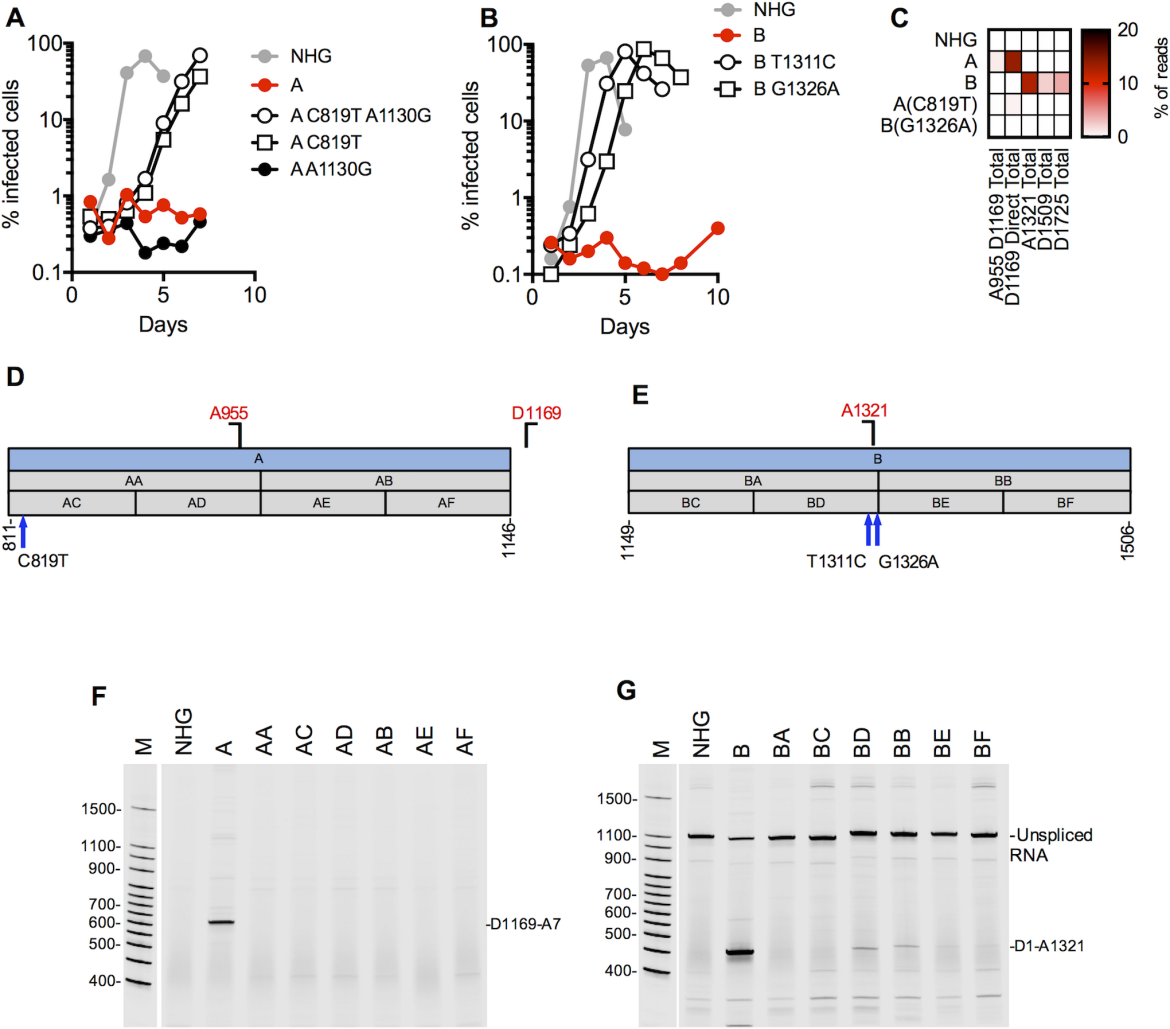
Activation of cryptic splice sites by synonymous mutations in Gag. (A, B) MT4 cells were infected with the indicated virus (harvested from the supernatant of 293T cells transfected proviral plasmids representing each of the WT(HIV-1_NHG_), mutant (A, B and revertants (A C819T, A1130G and B T1311C, G1326A) thereof) at an MOI of 0.002. Aliquots of infected cells were withdrawn each day and the proportion of infected cells determined by FACS analysis of GFP expression. (C) Next gen sequencing analysis of HIV-1 splicing. The heatmap indicates relative proportion of sequencing reads that used the cryptic splice sites for WT(HIV-1_NHG_), mutant (A, B and revertants thereof). (D,E) Schematic representation of the mutant blocks of nucleotides in HIV-1 mutants A (D) and B (F), indicating positions of mutant derivatives (AA, AB, BA, BB) etc, and the positions of cryptic splice sites and revertant mutant sites. Blocks colored blue are those that conferred overt splicing perturbations when mutated. (F) Fluorescent primer PCR analysis of HIV-1 splicing in mutant A. A sense PCR primer situated 5’ to the cryptic donor D1169, was used along with an antisense primer positioned 3’ to A7 (labelled with IRD800) was used to amplify cDNAs derived from the 1.8 kb class of spliced HIV-1 mRNAs respectively. (G) Fluorescent primer PCR analysis of HIV-1 splicing in mutant B. A sense PCR primer situated 5’ to D1, was used along with an antisense primer positioned 3’ to the mutant B block (labelled with IRD800) was used to amplify cDNAs derived HIV-1 mRNAs. For panels (F) and (G) PCR products were subjected to PAGE and a LI-COR Odyssey scanner was used to detect fluorescent signals directly from the gels.

For mutants A and B in which cryptic splice sites within the *gag* gene were activated by the silent mutations, we first attempted to derive revertant viruses by passage in MT4 cells. For both mutants A and B, passage quickly yielded viruses that replicated more rapidly than the parental mutants viruses (Fig 5A and 5B, S2 Fig). In the case of mutant A, passage in MT4 cells yielded a revertant virus that replicated well, albeit with delayed kinetics relative to WT HIV-1_NHG_ and contained two nucleotide substitutions relative to the A mutant parent. One of these mutations (C819T) was responsible for the revertant replication phenotype (Fig 5A). The C819T mutation was synonymous, and while it occurred at a position that differs from the WT in mutant A, the reversion was not to the WT sequence (WT = G819, mutant A = C819, revertant = T819). Thus, if position G819 in the WT virus was involved in a hypothetical RNA secondary structure that was perturbed or induced in mutant A (C819), then the C to T substitution in the revertant would not be expected to affect the perturbation of that secondary structure. The C819T revertant largely corrected the predominant splicing defects in mutant A, reducing the use of the cryptic splice acceptor (A_955_) from ~ 1% to ~0.1% and the cryptic splice donor (D_1169_) from ~14% to <1% (Fig 5C). Since the reversion mutation C819 was distal to the cryptic splice sites (~140 and ~350 nucleotides 5’ to A_955_ and D_1169_, respectively, Fig 5D) the mechanism by which it exerts its effect was unclear. Notably, the revertant mutation occurred within a few nucleotides of the reported secondary structure that includes the HIV-1 packaging sequence, and D1 (which is at position 743). Thus, it may be that the revertant mutation acts by modulating the secondary structure surrounding D1, rather than on the cryptic A_955_ and D_1169_ that were activated in mutant A.

For mutant B, passage in MT4 or CEM cells yielded two different replicating revertant viruses each of which contained a single nucleotide substitution relative to the B mutant (G1326A in MT4 cells and T1311C in CEM cells) (Fig 5B, S2 Fig) Both revertant mutations were synonymous, at positions that differed in WT and mutant B viruses. Both mutations were proximal to the cryptic splice acceptor (A_1321_) that was activated by the B mutations (Fig 5E).

Analysis of the G1326A revertant in the NGS splicing assay indicated reduced use of the cryptic acceptor A_1321_, D_1509_, and D_1725_ (from 12%, 2.9%, and 3.9% to <1% respectively Fig 5C). Given the proximity of the reversion mutations to the silenced cryptic splice site, it is likely that these mutations act directly to reduce splicing factor binding, and thereby reduce the use of the cryptic A_1321_. However, only the T1311C mutation had a marginal effect on the predicted strength of A_1321_, reducing the MaxEnt score from7.03 to 6.09 (S3 Table), while G1326A had no effect on the MaxEnt score, yet this mutation abolished the use of A_1321_ as an acceptor. Mutations in mutant B may have created or revealed a splicing factor binding site that was otherwise limiting for the use of the cryptic A_1321_ in a manner that was reversed by the G1326A and T1311C revertant mutations.

To map mutations in A and B that were responsible for activating cryptic splice sites D_1169_ and A_1321_ respectively, we generated a set of mutant viruses (AA, AB, AC, AD, AE and BA, BB, BC, BD, BE) that contained subsets of the synonymous mutations present in mutants A and B (Fig 5D and 5E). We also designed a fluorescent primer-based PCR-PAGE assay in which a PCR primers were positioned to conveniently and specifically monitor the major aberrant splicing event in mutants A and B which, as expected, yielded PCR product consistent with splicing at the respective cryptic splice sites (D_1169_ and A_1321_, respectively) (Fig 5F and 5G). Surprisingly, none of the secondary mutants containing subsets of the A and B mutations recapitulated the effects of the A and B mutations (Fig 5F and 5G). Thus, the activation of the cryptic splice sites by the Group 2a mutants A and B was the result of multiple synonymous changes in those mutant viruses. Additionally, the apparent inability of MaxEnt scoring to consistently predict cryptic site utilization in the context of these mutants indicated that splicing defects could not be due solely to direct enhancing effects of mutations on specific cryptic sites.

### Multiple features of HIV-1 RNA suppress splicing at A1 and A2

**Fig 6:**
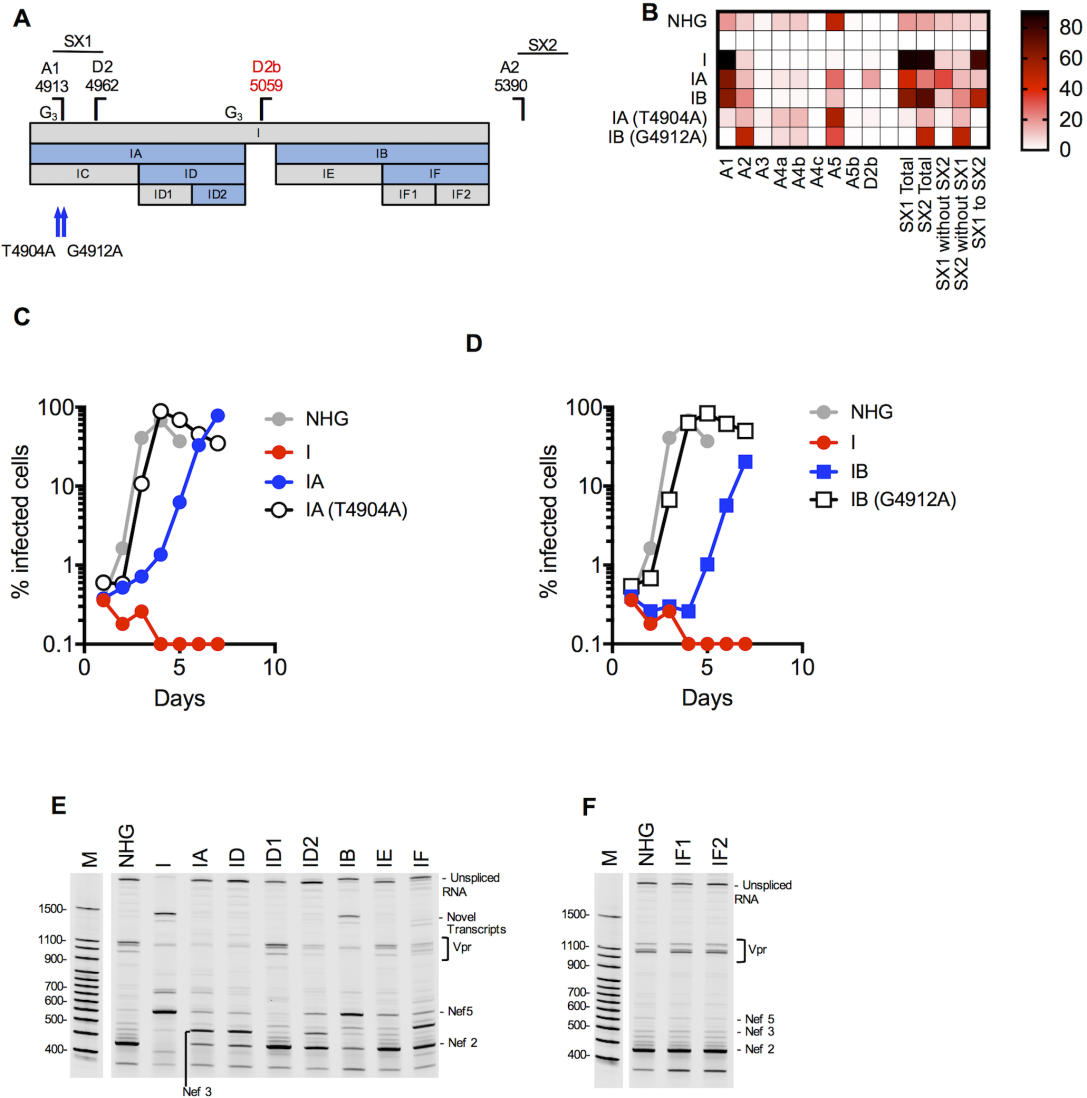
Activation of canonical splice acceptor sites (A1 and A2) by synonymous mutations in mutant I. (A) Schematic representation of the mutant blocks of nucleotides in HIV-1 mutant I, indicating positions of mutant derivatives (IA, IB, IC….etc), and the positions of splice sites and revertant mutant sites (blue arrows). Blocks colored blue are those that conferred overt splicing perturbations when mutated. (B) Next gen sequencing analysis of HIV-1 splicing in transfected 293T cells. The heatmap indicates relative proportion of sequencing reads that indicate direct splicing to the indicated acceptors or inclusion of the short exons (SX1 and/or SX2) as indicated at the bottom of the heatmap for WT(HIV-1_NHG_), mutants I, IA, IB and revertants thereof. (C, D) MT4 cells were infected with the indicated virus (harvested from the supernatant of 293T cells transfected proviral plasmids representing WT(HIV-1_NHG_), mutant (I, IA, IB and revertants thereof) at an MOI of 0.002. Aliquots of infected cells were withdrawn each day and the proportion of infected cells determined by FACS analysis of GFP expression. (E, F) Fluorescent primer PCR analysis of HIV-1 splicing. 293T cells were transfected with the indicated WT and mutant proviruses, RNA extracted and cDNA synthesized. A sense PCR primer situated 5’ to the major splice donor, was used along with an antisense primer positioned 3’ to A7 (labelled with IRD800) to amplify cDNAs derived from the 1.8 kb class of spliced HIV-1 mRNAs. PCR products were subjected to PAGE and a LI-COR Odyssey scanner was used to detect fluorescent signals directly from the gels. Salient mRNA species determined by direct sequencing of extracted gel bands, or inferred from Nextgen sequencing assays are indicated.

For mutant I, which exhibits overuse of A1 and A2, as well as the corresponding donors (D2 and D3) positioned immediately 3’ to A1 and A2, (Fig 6A) we failed to obtain revertant replication competent viruses, even after extended passage. Therefore, we divided the I segment into 5’ and 3’ halves and generated two derivative mutant viruses (IA and IB) each of which had approximately half the of the synonymous mutations that were present in I (Fig 6A). Both IA and IB mutants also exhibited splicing defects that were primarily manifested as overuse of A1, but these defects were less complete, in that some degree of direct splicing to downstream acceptors (e.g. A5) was present in both IA and IB (Fig 6B). Notably, IA exhibited oversplicing at A1 and (unlike the parent mutant I) the cryptic splice donor D2b [37]. Conversely IB exhibited direct oversplicing at both A1 and, to some degree, to A2 but not D2b (Fig 6B).

Both IA and IB could replicate with delayed kinetics compared to HIV-1_NHG_, and extended passage of IA and IB yielded point mutation revertants that could replicate with kinetics close to those of HIV-1_NHG_ (Fig 6C and 6D, S2 Fig). In the case of IA, a T4904A mutation occurred in the A1 polypyrimidine tract (Fig 6A and 6C). This caused a profound reduction of splicing at A1 and likely as a consequence, reduction of the inclusion of SX1 (A1-D2) in spliced RNAs (Fig 6B). Use of the cryptic D2b site was also abolished in the IA(T4904A) revertant. In fact, other than underuse of A1 and reduced inclusion of SX1, the IA(T4904A) revertant had a near normal splicing pattern Thus, activation of canonical and cryptic downstream splice donors in mutant IA appeared to be secondary to activation of A1.

For mutant IB, the situation was more complex; a revertant (G4912A) was recovered after passage (S2 Fig), precisely at the A1 acceptor that abolished the use of A1, and consequently the inclusion of SX1 into spliced mRNA (Fig 6A, 6B and 6D). However, significant overuse of A2, and consequent inclusion of SX2 (A2-D3) remained evident in the IB(G4912A) revertant (Fig 6B). Indeed, overuse of A2 was more prominent in the IB(G4912A) revertant than in IB, perhaps because of the functional removal of A1 (Fig 6B). It was therefore apparent that native sequences within IA result in suppression of splicing at A1, while sequences within IB cause suppression of splicing at both A1 and A2.

To map elements within IA and IB that control splicing at A1 and A2, we used the fluorescent PCR-based assay to analyze the pattern of splicing for viruses containing subsets of the IA and IB mutations. For IA, analysis of viruses containing smaller component mutant elements (IC and ID, Fig 6A) revealed that aberrant splicing was conferred only by the ID element (Fig 6E). Thereafter, when ID was subdivided into ID1 and ID2, it was evident that the controlling element resided primarily within ID2 (Fig 6E). Thus, this analysis revealed a 48 nucleotide sequence that appeared to suppress splicing at A1. Notably, a novel ESS/ESE element, termed ESS2b/ESE2b was recently identified that nearly precisely coincides with ID2 [32], indicating that our approach has the potential to identify novel splicing regulatory signals.

Notably, the unmutated IA segment contained short runs of three G’s (G_3_) that have been reported to constitute hnRNP binding sites which suppress the activation of the cryptic splice donor D2b [37]. The mutations in IA disrupted two of these G_3_ runs (Fig 6A) and D2b was used in ~17% of IA spliced reads. The revertant mutation IA(4904) was able to inhibit activation of D2b from 17% to 2% (Fig 6B), suggesting that the activation of D2b is predominately a secondary effect of A1 overutilization. Nevertheless, one of the G_3_ motifs coincides with the ID2 segment and therefore disruption of the G_3_ motifs may also contribute to the activation of the cryptic D2b site.

For IB the situation was more complex, as mutations in this segment control splicing at both A1 and A2. Nevertheless, subdivision of the IB mutations into components IE and IF (Fig 6A), revealed that the majority the effect of IB mutations were conferred by mutations in IF. However, IF did not exhibit as prominent a degree of perturbation as IB (Fig 6E). Even though the splicing of mutant IE appeared normal, mutations in IE made some contribution to the defects present in IB (Fig 6E). Subdivision of IF into IF1 and IF2 yielded viruses with a normal pattern of splicing (Fig 6F). Thus, it was evident that multiple sequences acting together in IB, distributed over IE, but primarily concentrated in IF1 and IF2, regulate splicing at A1 and A2 and their overall contributions could not be mapped through this approach to a single small candidate regulatory element.

**Fig 7:**
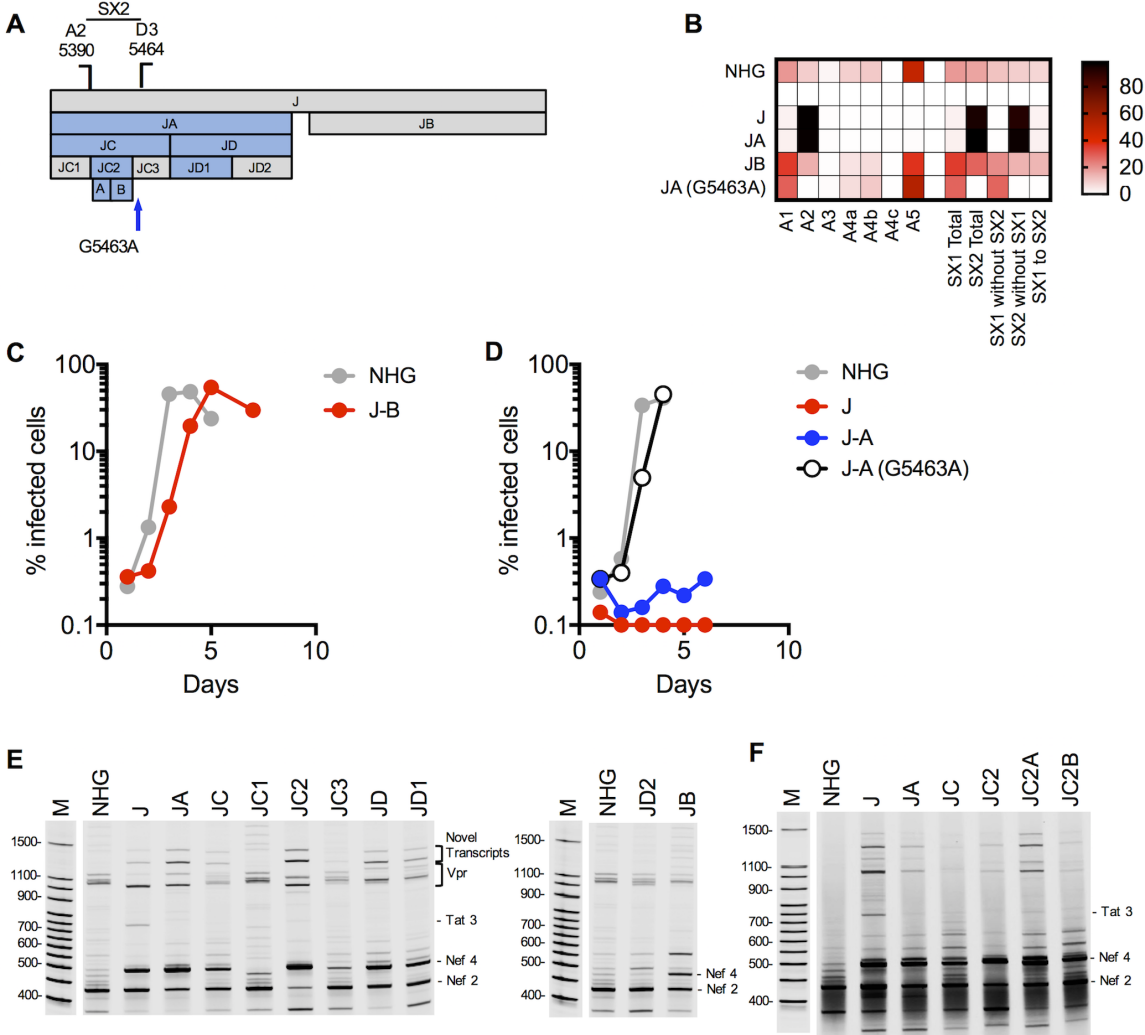
Activation of canonical splice acceptor site A2 by synonymous mutations in mutant J. (A) Schematic representation of the mutant blocks of nucleotides in HIV-1 mutant J, indicating positions of mutant derivatives (JA, JB, JC….etc), and the positions of splice sites and revertant mutant sites (blue arrows). Blocks colored blue are those that conferred overt splicing perturbations when mutated. (B) Next gen sequencing analysis of HIV-1 splicing in transfected 293T cells. The heatmap indicates relative proportion of sequencing reads that indicate direct splicing to the acceptors or inclusion of the short exons (SX1 and/or SX2) indicated at the bottom of the heatmap for WT(HIV-1_NHG_), mutants J, JA, JB and revertants thereof. (C, D) MT4 cells were infected with the indicated virus (harvested from the supernatant of 293T cells transfected proviral plasmids representing each of the WT(HIV-1_NHG_), mutant or revertant viruses at an MOI of 0.002. Aliquots of infected cells were withdrawn each day and the proportion of infected cells determined by FACS analysis of GFP expression. (E, F) Fluorescent primer PCR analysis of HIV-1 splicing. 293T cells were transfected with the indicated WT (HIV-1_NHG_) and mutant proviruses, RNA extracted and cDNA synthesized. A sense PCR primer situated 5’ to the major splice donor, was used along with an antisense primer positioned 3’ to A7 (labeled with IRD800) to amplify cDNAs derived from the 1.8 kb class of spliced HIV-1 mRNAs. PCR products were subjected to PAGE and a LI-COR Odyssey scanner was used to detect fluorescent signals directly from the gels. Salient mRNA species determined by direct sequencing of extracted gel bands, or inferred from Nextgen sequencing assays are indicated.

For mutant J, which exhibited overuse of A2 and D3 we also failed to obtain revertant replication competent viruses, even after extended passage. Therefore, we divided the J segment into 5’ and 3’ halves and generated two derivative mutant viruses (JA and JB, Fig 7A). Although there was some degree of splicing perturbation in JB (Fig 7B), this perturbation was modest compared to J and JA. Moreover, JB was only marginally delayed in spreading replication assays compared to HIV-1_NHG_. (Fig 7C). Therefore, we did not attempt to select JB revertants. Conversely, JA recapitulated the splicing perturbation observed in J, and was replication defective (Fig 7B and 7D). Extended passage of mutant JA yielded a revertant mutation (G5463A) that enabled replication (Fig 7D, S2 Fig). Notably, the reversion mutation was precisely at, and inactivated donor D3 (Fig 7A), but also abolished splicing to A2 (Fig 7B). The enhancing effect of D3 on A2 splicing has previously been demonstrated [38]. Interestingly, even though D3 appeared to be required for splicing at A2 only a fraction of RNAs that are spliced to A2 are also spliced at D3. It was notable that the JB mutants as well as the JA(G5463A) revertant exhibited some oversplicing to A1 (and therefore elevated inclusion of SX1) even though the J mutant sequences were distal (~440 to 890 nucleotides) to A1 (Fig 7A and 7B).

To map elements within JA that control splicing, we used the fluorescent PCR-based assay for the 1.8 kb HIV-1 mRNAs to analyze viruses containing subsets of the JA mutations. We divided the JA mutant segment into 5’ and 3’ halves in two derivative mutant viruses (JC and JD, Fig 7A) which both exhibited some degree of perturbed splicing (Fig 7E). Further subdivision of JC into JC1, JC2 and JC3 clearly suggested that the 20 nucleotide JC2 segment contained an element whose mutation was primarily responsible for the perturbed splicing in JC (Fig 7E), but further division of 20 nucleotide JC2 yielded two mutant segments (JC2A and JC2B) both of which cause perturbed splicing to nearly the same degree as the J, JA, JC and JC2 mutant segments from which they were derived (Fig 7F). Division of the JD segment into JD1 and JD2 clearly revealed another element within the 46 nucleotide JD1 segment, that when mutated yielded a oversplicing pattern similar to that of the J mutant virus (Fig 7E). Thus, multiple mutations within the JA fragment, contained within the segments JC2 and JD1 were capable of causing oversplicing defects similar to those observed in the J mutant.

### A discrete RNA sequence regulates HIV-1 splicing at A3.

**Fig 8:**
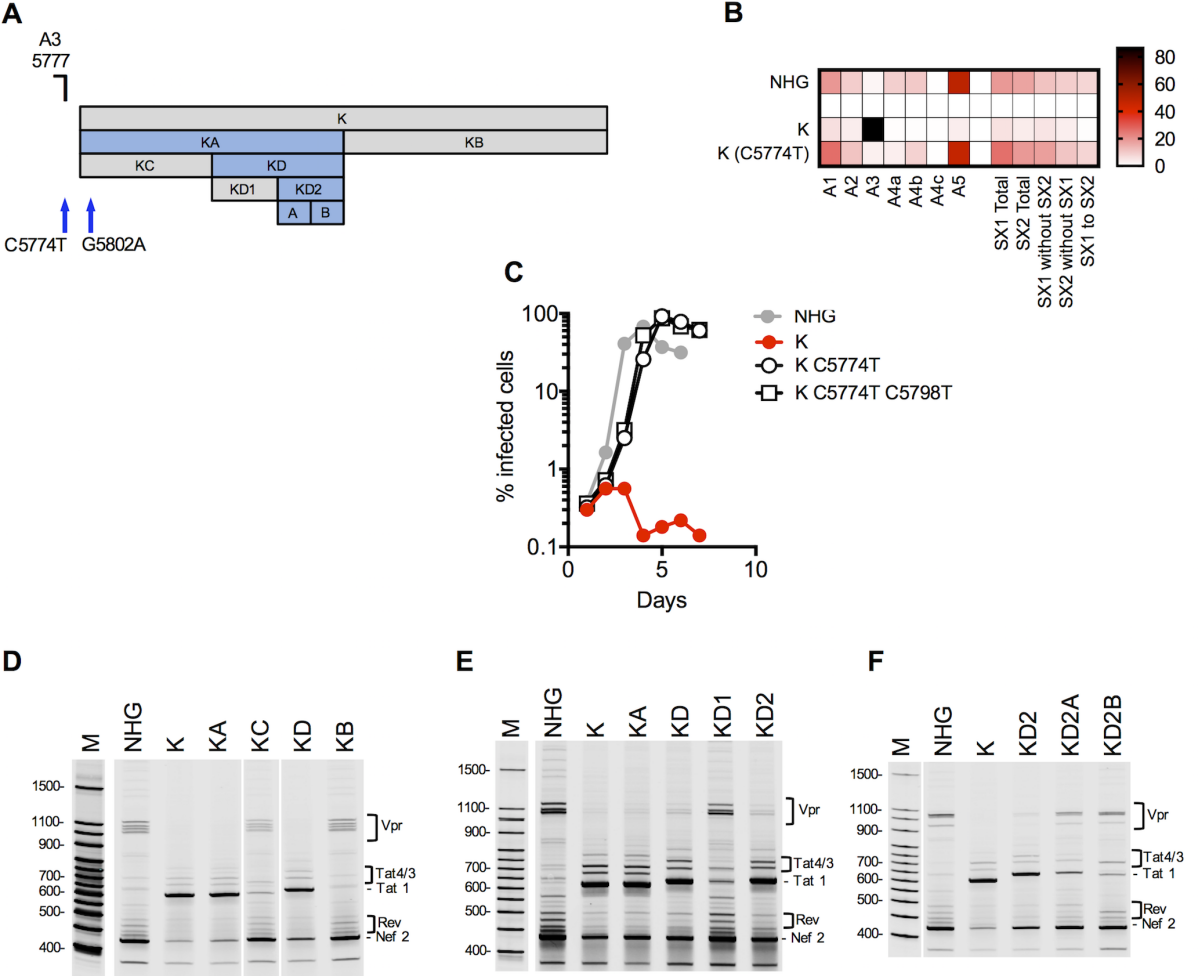
Activation of canonical splice acceptor site A3 by synonymous mutations in mutant K. (A) Schematic representation of the mutant blocks of nucleotides in HIV-1 mutant K, indicating positions of mutant derivatives (KA, KB, KC….etc), and the positions of splice sites and revertant mutant sites (blue arrows). Blocks colored blue are those that conferred overt splicing perturbations when mutated. (B) Next gen sequencing analysis of HIV-1 splicing in transfected 293T cells. The heatmap indicates relative proportion of sequencing reads that indicate direct splicing to the acceptors or inclusion of the short exons (SX1 and/or SX2) indicated at the bottom of the heatmap for WT(HIV-1_NHG_), mutant K and the C5774T revertant. (C) MT4 cells were infected with the indicated virus (harvested from the supernatant of 293T cells transfected proviral plasmids representing each of the WT(HIV-1_NHG_), mutant or revertant viruses at an MOI of 0.002. Aliquots of infected cells were withdrawn each day and the proportion of infected cells determined by FACS analysis of GFP expression. (D, E, F) Fluorescent primer PCR analysis of HIV-1 splicing. 293T cells were transfected with the indicated WT (HIV-1_NHG_) and mutant proviruses, RNA extracted and cDNA synthesized. A sense PCR primer situated 5’ to the major splice donor, was used along with an antisense primer positioned 3’ to A7 (labeled with IRD800) to amplify cDNAs derived from the 1.8 kb class of spliced HIV-1 mRNAs. PCR products were subjected to PAGE and a LI-COR Odyssey scanner was used to detect fluorescent signals directly from the gels. Salient mRNA species determined by direct sequencing of extracted gel bands, or inferred from Nextgen sequencing assays are indicated.

For mutant K (Fig 8A), which exhibits overuse of A3 (Fig 8B), it proved straightforward to recover a revertant mutant virus through passage that corrected the splicing defect and replicated well (Fig 8B and 8C, S2 Fig). This revertant contained two mutations, one of which (C5774T) was sufficient to restore replication to near WT kinetics (Fig 8C). This functional reversion mutation was 3 nucleotides from A3. Notably the K(C5774T) revertant not only corrected overuse of A3 but exhibited a splicing pattern that was nearly indistinguishable from that of HIV-1_NHG_ (Fig 8B). This was surprising because previous studies have reported that CAG and TAG are used at a similar efficiency as 3’ splice acceptors in the context of HeLa cell nuclear extracts [39]. The remarkable diminution of splicing in the K(C5774T) reversion mutant suggests that in the context of A3, the TAG is far less well utilized than CAG. Further, the A3 MaxEnt score of is increased from 9.76 to 10.05 in the context of the K(C5774T) reversion mutant, predicting that A3 is a stronger acceptor in K(C5774T). However, experimentally the reverse is the case, demonstrating the limitation of both *in silico* and *in vitro* analyses to predict splicing phenotypes in the context of a full-length HIV-1 construct in a living cell.

To map sequences within the K mutant that were responsible for causing oversplicing, we divided the K mutant segment into two halves (KA and KB, Fig 8A) and analyzed the pattern of 1.8 kb mRNAs using the fluorescent primer PCR assay. This analysis revealed that mutations responsible for A3 overuse resided in KA (Fig 8D). Then, further subdivision of KA (into KC and KD) revealed that KD contained the controlling element(s) (Fig 8D). Finally, subdivision of KD (into KD1 and KD2) showed that KD2 contained RNA sequences whose mutation caused oversplicing (Fig 8E), but further subdivision of KD2 (into KD2A and KD2B) showed that mutations in both of these KD2 components contributed its effect (Fig 8F). Thus, a 23-nucleotide element (KD2) positioned >100nt from A3 contained an RNA element whose sequence influences splicing at A3.

## Discussion

Through a global synonymous mutagenesis experiment we found that extensive portions of the HIV-1 genome could be synonymously mutated without major effects on viral replication (Group 1 mutants). In particular, synonymous mutations throughout the majority of the *pol* gene had a minimal or no effect on viral fitness, such that their effect was not measurable in our assays. Given the extent of the mutations that were introduced, and the improbability that most RNA secondary structures would be preserved in our mutants, it seems unlikely that undiscovered specific RNA secondary structures essential for replication exist in portions of the viral genome covered by Group 1 mutants. Even among mutants that were replication defective, Group 2 mutants could be restored to replication competence through single nucleotide reversion mutations that suppressed the utilization of cryptic or canonical splice sites, whose use was enabled or elevated in the global mutagenesis experiment. Again, this argues against the notion that undiscovered, specific RNA structures that are essential for replication are prevalent in Group 2 mutants, with the exception of those that regulate splicing.

An important caveat to this conclusion is that replication was measured in permissive cell lines in the absence of competition. It is possible, even likely, that mutants or revertants with WT or near WT replication dynamics, have modest fitness deficits that would be evident in a more stringent environment or a competitive replication assay. For example, some of the HIV-1 accessory genes are important *in vivo*, but non-essential *in vitro*, therefore defects in their expression would be expected to have minimal effects on replication in our assays. Thus, while we can reasonably conclude that RNA secondary structures in Group 1 and Group 2 revertant mutants are non-essential for replication *per se*, it is nevertheless possible that RNA structures play an accessory role in regulating or fine-tuning the levels or fates of mRNAs encoding certain accessory proteins.

Nevertheless, synonymous mutations in some portions of the HIV-1 genome caused profound, near-lethal defects in these highly permissive T-cell lines (Group 2 and Group 3 mutants). These mutations therefore perturbed essential, non-coding features of the HIV-1 nucleotide sequence. One noncoding feature of the HIV-1 genome that appeared important for replication that was uncovered by our Group 2a mutants was suppression of cryptic splice sites near the 5’ end of the RNA genome. For these mutants, the magnitude of the deviation from WT sequence appeared important for cryptic site activation. Simply subdividing the mutant sequence blocks into two mutant blocks approximately equal lengths (e.g. mutants AA, AB and BA, BB) reverted the mutant splicing phenotype. This finding suggests that activation of the existing cryptic splice sites resulted from multiple perturbations to the *gag* nucleotide sequence and that changes in predicted splice site strength were not sufficient to explain their activation. Moreover, in the case of mutant A, a single nucleotide revertant mutation that occurred distal to the activated cryptic splice sites corrected the splicing defect without affecting their MaxEnt scores. These findings might be best explained by the existence of multiple elements in the *gag* gene (secondary structures or protein binding sites) that act redundantly to suppress cryptic splice site utilization. The fact that the revertant mutation for mutant A occurred at a position proximal to an existing RNA structure that includes D1 [40, 41], may suggest a role for an extended secondary structure involving the 5’ leader and the *gag* gene in suppressing the utilization of potential cryptic splice sites in *gag*. The potential for the 5’ leader to form alternative structures that could affect splicing appears to be finely tuned [42], and therefore could possibly be perturbed by distal mutations in *gag*. Clearly, further work will be required to understand how the WT noncoding RNA sequence is selected to avoid utilization of cryptic splice sites.

**Fig 9:**
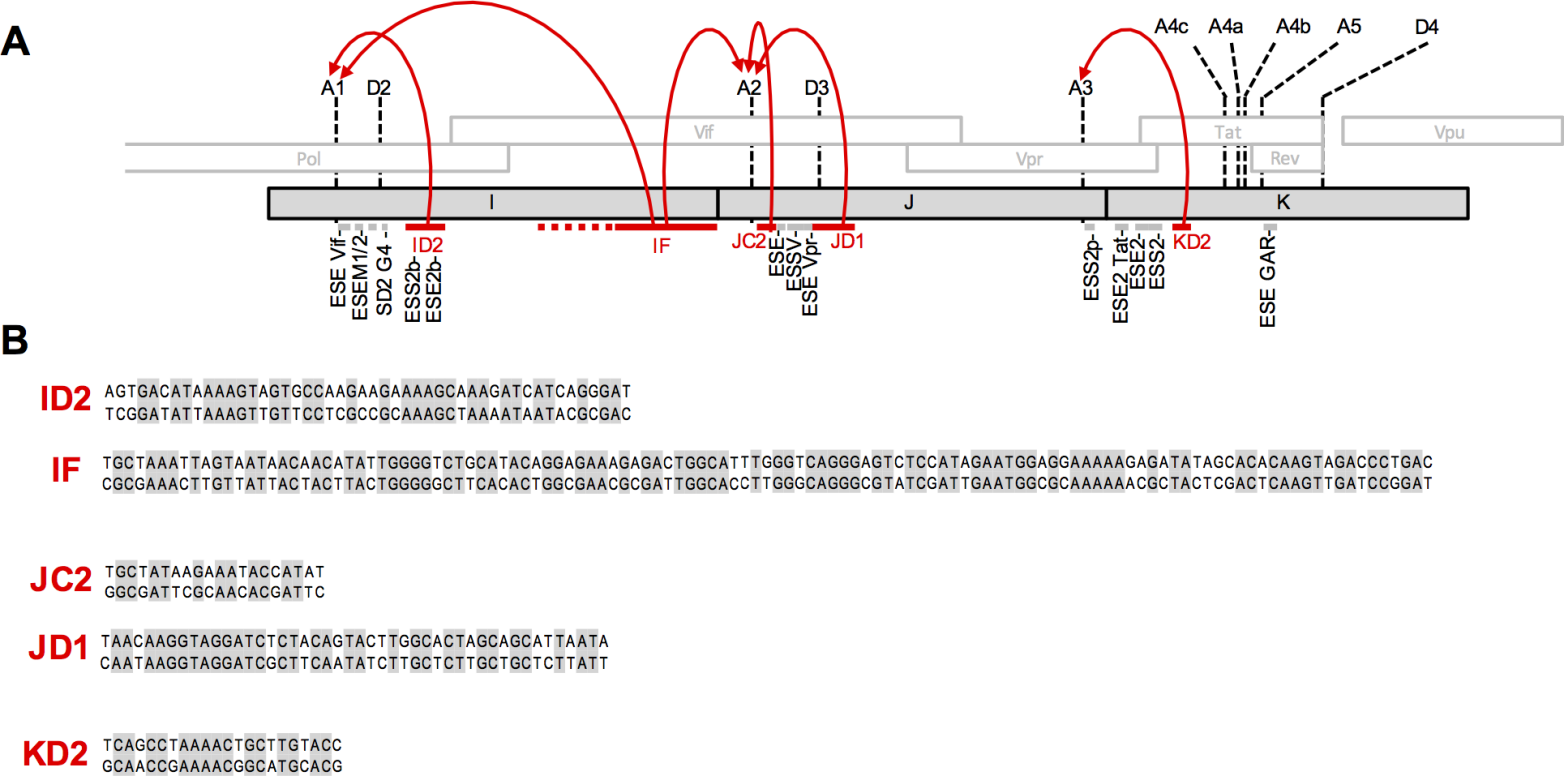
Summary of splicing control in HIV-1. (A) Schematic representation of the central portion of the HIV-1 genome with the positions of canonical splice sites indicated. Previously identified splicing control elements are indicated with grey lines. Sequences identified in this study whose mutation enhanced splicing are indicated with red lines and the splice acceptors on which they act are indicated with red arrows. (B) The sequences of the identified elements that affect splicing (upper line = WT sequence, lower line = oversplicing mutant sequence) are shown.

A detailed analysis of Group 2b mutants, that targeted the central portion of the genome, revealed several elements whose perturbation resulted in dramatic overuse of the canonical splice acceptors A1, A2, and A3. These mutant elements that resulted in oversplicing could be mapped to sequences of various lengths. Such sequences constitute candidate individual or clustered ESS elements. When combined with existing knowledge of splicing regulation, at A1, A2 and A3 [4, 21, 23–32], these and previous findings suggest a highly complex regulatory network of functional inputs that govern alternative splicing of the HIV-1 genome, as depicted in (Fig 9).

The phenotypes of some mutant and revertant viruses (i.e. viruses with perturbed splicing whose replication was recovered by mutations at splice sites) is consistent with the notion that exon definition (i.e. coordinated recognition of a 5’ splice acceptor and a 3’ splice donor by the splicing machinery [43]) plays a central role in the regulation of HIV-1 alternative splicing. For example, the mutant (IA) that exhibits elevated use of A1 and the downstream donor D2 (as well as the cryptic donor D2b) while the revertant (IA(T4904A)) occurred at A1 and reduced utilization of D2, and D2b as well as A1. Similarly, in IB, that exhibits elevated use of both A1 and A2, both downstream donors D3 and D2 are also overused. In this case, the revertant mutation (IB(G4912A)) at A1 caused abolition of splicing at both A1 and D2 while splicing at A2 and D3 remain elevated. Consistent with the notion that splicing at A1 and D2 are coordinated, previous work has shown that the efficiency with which D2 is recognized can affect the frequency of splicing at A1 [44]. Similar communication occurs between A2 and D3. Indeed, for a mutant (JA) that exhibited profound oversplicing at A2 and D3, a reversion mutation (JA(G54653A)) that inactivated D3 also caused abolition of splicing at A2. Because not all splices at A2 lead to splicing at D3, this observation suggests that recognition of D3 by the splicing machinery is required for splicing at A2 even when D3 is not utilized, as has previously been suggested [23, 38]. Overall therefore, a major determinant of the use of a given acceptor or donor in the HIV-1 genome was the use of a downstream donor or upstream acceptor, respectively.

Our data is also consistent with the notion that splice acceptor sites compete with each other, with ‘cascading’ effects based on exon definition. For example, the mutant (JA) under-utilizes A1 and over-utilizes A2. Its revertant (JA(G54653A)) that abolishes splicing at both D3 and A2, causes over-utilization of A1. In a reciprocal example of A1-A2 competition, abolition of A1 utilization in the revertant IB(G4912A), was accompanied by increased splicing at A2. Acceptor competition was also evident in the context of the mutant K, which exhibited oversplicing at A3 at the expense of splicing at A1, A2, A4b and A5. In this case, the reversion mutation at A3 in K(C5774T) led to restoration of WT splicing frequency at all other sites in the central portion of the HIV-1 genome.

Overall therefore, a key regulator of the use of a particular splice site is the presence and utilization of other splice sites, through coordinated recognition of acceptor and donor sites, along with competition between acceptor sites. Thus, disruption of the splicing regulatory signals whose existence is indicated herein can have complex effects on overall splicing, through the propagation of their effects from one splice site to another. Clearly the overall effect of these perturbations is best appreciated in the nextgen sequencing assay with all splice sites represented in a viral construct.

That being the case, the RNA sequence elements that we have identified as apparent regulators of splice acceptor utilization (Fig 9) could work directly or indirectly. Specifically, they could act to directly inhibit access of the splicing machinery to the affected splice acceptors or indirectly through splice donors, inhibiting acceptor utilization by inhibiting exon definition. It is also possible that our mutations affect the ability of splice sites to effectively communicate with each other in the context of exon definition. Existing splicing regulatory sequences have been reported to exert their effect through binding of hnRNP or SR proteins, or through the formation of RNA secondary structures [45]. The sequences that we have identified are of varying sizes; some elements that had major effects on splicing appear sufficiently small (e.g. JC2 and KD2) to constitute specific protein binding sites. However, these elements did not appear to be enriched in canonical hnRNP binding motifs, as might be expected for splicing silencers [45]. Some of the effects on splicing that we found in our mutants, particularly within the I and J fragments, appeared complex and not easily mapped to small discrete elements. Perhaps these effects are the result of combinatorial inputs from multiple binding sites or secondary structures that could act to occlude splice sites, or spatially separate 3’ donors from 5’ acceptors thereby inhibiting exon definition. Further work will be required to determine precisely how these elements inhibit HIV-1 splicing.

Perturbation of balanced splicing did not always lead to abolition of HIV-1 replication. For example, the mutants JB and JA(5463A) had perturbed splicing (overuse of A1 for JA, and abolition of SX2 utilization for JB) but their replication was only slightly delayed compared to WT. Similarly the IB(G4912A) revertant virus had near WT replication but completely lacked splicing to A1 and therefore abolished inclusion of SX1. These perturbations would be expected to lead to underexpression or overexpression of Vif and Vpr, neither of which are essential for replication in the particular cell type used in our experiments. Thus, the requirement for a particular balance of HIV-1 mRNAs could be highly context dependent. In our experiments, replication defects that resulted from over-splicing were likely the result of depletion of the pool of unspliced RNA, thus leading to lower levels of synthesis of Gag, Pro, Pol, and other viral proteins, and lower levels of viral genomes for packaging. Thus, a key role of splicing inhibitory signals in the HIV-1 genome is to maintain the unspliced RNA pool, as well as adequate levels of necessary viral proteins.

Overall, our global synonymous mutagenesis experiment has revealed several RNA elements whose native sequence is important for HIV-1 replication. In particular, we have identified several RNA elements in the HIV-1 genome whose native sequence appears to be important for suppression of canonical splice sites, regulation of alternative splicing and maintenance of unspliced transcript levels. Additionally, our analysis revealed that some as yet unidentified property of RNA sequences in the *gag* gene suppresses utilization of cryptic splice sites. Understanding how RNA sequence affects splicing in the context of HIV-1 may give insights into the general mechanisms by which alternative splicing is regulated and how splicing regulation evolves, as well as opportunities to intervene therapeutically in HIV-1 infection.

## Material and methods

### Cell lines, viruses and infections

293T cells (ATCC) were grown in DMEM with 10% fetal bovine serum (Sigma). MT4 cells (NIH AIDS Reagent Repository) were cultured in RPMI supplemented with 10% fetal bovine serum. HIV-1_NHG_ and mutant derivatives containing a reporter GFP in place of *nef* were produced by transfection of 293T cells with the proviral plasmid using PEI. Virus titers were determined by FACS analysis of target MT4 cells. Dextran sulfate was added to inhibit reinfection at 18 hours post infection and the number of infected cells determined by FACS analysis of GFP expression 2 days post infection.

For spreading replication infections, 2x10^5^ MT4 cells in 2 mL of media were infected at an MOI of 0.002. Aliquots of infected cells were withdrawn each day, fixed in 4% PFA and the proportion of infected cells determined by FACS analysis of GFP expression. For single cycle infections, cells were infected with HIV-1_NHG_ or mutants thereof, at an MOI of 1.0, and harvested 2 days post infection for western blot and qPCR analysis. Serial passage of mutant viruses was started by infecting a culture of 5x10^5^ MT4 cells at an MOI of 0.002 and time points were fixed in 4% PFA. Upon infection of the entire culture, 200 μL of cell free supernatant was filtered and used to inoculate a new culture of 5x10^5^ MT4 cells. After the final passage, the cells were collected and the DNA was extracted (Qiagen Tissue Mini Kit), then the mutated region was sequenced to determine the revertant mutation that had occurred.

### Construction of mutant proviral plasmids

The mutated regions of the HIV-1 genome were synthesized (Genewiz) and cloned into HIV-1_NHG_ using restriction digest sites that were proximal to the mutated regions (S2 Table). Division of the original mutants blocks into two new derivative mutants was achieved using overlap extension PCR based approaches with mutant and WT templates. Revertant mutations acquired through passage of the virus were reconstituted into the original mutant provirus from which they arose through site directed mutagenesis and overlap extension PCR.

### PCR quantification of unspliced HIV-1 RNA

RNA was extracted from infected cells using Trizol and reverse transcribed using the SuperScript III reverse transcriptase (ThermoFisher). The resulting cDNA was used as a temple for quantitative real-time PCR using the ABI Fast RT-PCR system along with the Fast Start TaqMan Probe master mix. Unspliced viral RNA was detected using a forward primer: 5’-GGACTTGAAAGCGAAAGGGA-3’, a reverse primer: 5’-TCTCTCTCCTTCTAGCCTCCG-3’ and a TaqMan probe 5’-GGGCGGCGACTGGTGAGT-3’ targeting the major splice donor D1. Serial tenfold dilutions of known copy numbers of HIV-1_NHG_ plasmid was used to generate a standard curve.

### Antibodies and western blotting

Cells were normalized for cell number, lysed in SDS sample buffer, separated by electrophoresis on NuPage 4–12% Bis-Tris gels (Novex) and blotted onto nitrocellulose membranes (GE Healthcare). Blots were probed with an HIV-1 anti-capsid antibody (183-H12-5C) obtained from the NIH AIDS reagent repository, a GFP antibody (G1546, Sigma), and HIV-1 anti-Env gp120 antibody (12-6205-1, American Research Products).

### Analysis of HIV-1 splicing with fluorescent primer PCR

RNA from 293T cells transfected with mutant provirus was extracted using the Nucleospin RNA extraction kit (Machery Nagel). RNA was reverse transcribed using SuperScript III reverse transcriptase (ThermoFisher) with gene specific primers for either fully spliced (8483R: 5’-CCGCAGATCGTCCCAGATAAG-3' and partially spliced (6223R: 5'-CAAGTGCTGATATTTCTCCTTCAC -3') mRNA classes. The cDNA templates were then used in a 10μL PCR reaction with fluorescent reverse primers specific to the splice class (labelled at their 5’ ends with IRD800) and a forward primer position 5’ to the major splice donor (499F: 5' -CTGAGCCTGGGAGCTCTCTGGC-3') and run for 25 cycles with an annealing temperature of 54°C. Alternatively, to determine use of the activated cryptic splice site in mutant A, a forward primer, positioned 5’ to the mutations (763F 5’- TGACTAGCGGAGGCTAGAAGGAGAGAG -3’) and the fluorescent reverse primer for the fully spliced class (8483R) were used in a PCR reaction with the cDNA templates. To determine use of the activated cryptic splice site in mutant B, the forward primer 499F and a fluorescent reverse primer 5’ to the mutations in B (1557R 5’- GATAGGTGGATTATGTGTCATCC -3’) were used in a PCR reaction with the cDNA template. Then, 10μL of 2x TBE-Urea sample buffer was added to the PCR reaction which was then run on a 6% TBE-Urea gel for 90 minutes at 180V (Novex). A LI-COR Odyssey scanner was used to detect fluorescent signals directly from the gels.

### Analysis of HIV-1 splicing using Primer ID-based deep sequencing

Determination of splice site utilization using the Primer ID-based deep sequencing assay was done substantially as described with minor modifications [20]. Briefly, RNA was extracted from cells transfected with HIV-1_NHG_ or mutants thereof using the RNeasy Plus minikit (Qiagen). Primers used for cDNA synthesis were GTGACTGGAGTTCAGACGTGTGCTCTTCCGATCTNNNNNNNNNNNNNNCAGTTCGGGATTGGGAGGTGGGTTGC for 1.8 kb spliced transcripts and GTGACTGGAGTTCAGACGTGTGCTCTTCCGATCTNNNNNNNNNNNNNNGCTACTATTGCTATTTGTATAGGTTGCATTACATG for 4 kb spliced transcripts. Indexed primers were obtained from Integrated DNA Technologies Custom Oligos. Total cell RNA (8 μg) was subjected to cDNA synthesis, purification and cleanup and an initial PCR amplification. An aliquot of the product from this first PCR was used as the template for the second PCR to add the Illumina adapter and bar codes to allow multiplexing in the Illumina sequencing reaction. PCR products were visualized on a 2% agarose gel and then cleaned. Libraries were mixed/multiplexed and sequenced using the 300-base paired-end read for the Illumina Miseq platform. Reads were sorted using the Illumina bcl2fastq pipeline (v.1.8.4) to separate the multiplexed samples. Subsequent Data analysis and splicing quantification were done using the previously described in-house pipeline [20] written in Ruby and adapted to accommodate the mutated sequences. This program uses the combined sequence information from the paired-end reads to identify splice site usage and transcript type. Reads are condensed by Primer ID to prevent skewing in the PCR steps. Cryptic alternative donor and acceptor splice sites were identified using a program that compares data reads to a reference sequence and identifies the base where a splice discontinuity occurs and the base it splices to.

## Supporting information

S1 Table Sequences containing known cis-acting elements left undisturbed in synonymously mutated viruses. (DOCX)

S2 Table Positions of sequences targeted in synonymously mutated HIV-1. (DOCX)

S3 Table MaxEnt scores of splice acceptors and donors in wild type and mutated sequence. (DOCX)

S1 Data Fasta file containing sequences of WT HIV-1_NHG_ and synonymously mutated segments (A through P) aggregated into a single sequence. See S2 Table for coordinates of each mutant segment.
(TXT)

S2 Data Alignment of WT HIV-1_NHG_ and synonymously mutated derivatives. Excel file listing HIV-1_NHG_ nucleotide position, WT and synonymously mutated nucleotide sequence (A though P aggregated into a single sequence), open reading frames, positions of each mutant, splice acceptors and donors, splicing regulatory elements, and other RNA elements.
(XLSX)

S1 Fig Spreading replication properties of mutant viruses in CEMx174 cells. (A-P) MT4 cells were infected with the indicated virus (harvested from the supernatant of 293T cells transfected with each of the WT(HIV-1_NHG_) mutant (A-P) proviral plasmids at an MOI of 0.002. Aliquots of infected cells were withdrawn each day, fixed in 4% PFA and the proportion of infected cells determined by FACS analysis of GFP expression.
(TIFF)

S2 Fig Spreading replication experiments to recover second-site revertants of defective mutants. MT4 cells were infected with the mutant viruses (A, B, IA, IB, JA, K, as indicated, harvested from the supernatant of 293T cells transfected with each of the indicated mutant proviral plasmids). Aliquots of infected cells were withdrawn each day, fixed in 4% PFA and the proportion of infected cells determined by FACS analysis of GFP expression.
(TIFF)
