# Supplementary material for: Global synonymous mutagenesis identifies cis-acting RNA elements that regulate HIV-1 splicing and replication
Source: PLoS Pathog. 2018 Jan 29;14(1):e1006824. doi: 10.1371/journal.ppat.1006824 (PMC5805364; doi:10.1371/journal.ppat.1006824)
Supplement: S1 Table — (DOCX) [file ppat.1006824.s001.docx]

**S1 Table. Sequences containing known cis-acting elements left undisturbed in synonymously mutated viruses**

| **Site name** | **Position^1^** | **Reference** |
| --- | --- | --- |
| **Splice sites^2^** |  | Reviewed in[21, 23] |
| D1 | 744 |  |
| A1 | 4911 |  |
| D2 | 4963 |  |
| D3 | 5464 |  |
| A2 | 5388 |  |
| A3 | 5775 |  |
| A4c | 5935 |  |
| A4a | 5952 |  |
| A4b | 5958 |  |
| A5 | 5974 |  |
| D4 | 6045 |  |
| A7 | 8373 |  |
| **Splicing regulators** |  |  |
| ESS2b^3^ | 4988-5007 | [32] |
| ESSV | 5422-5436 | [45] |
| ESS2 | 5846-5855 | [46] |
| ESS3 | 8450-8465 | [47] |
| ESS2P | 5779-5785 | [25] |
| ESE2b^3^ | 5008-5032 | [32] |
| ESE vpr^3^ | 5437-5460 | [38] |
| ESE tat^3^ | 5807-5837 | [48] |
| (GAA)3 | 8418-8428 | [49] |
| ESE2 | 5838-5845 | [50] |
| ESEM1 | 4933-4939 | [28] |
| ESEM2 | 4956-4962 | [28] |
| ESE vif | 4918-4927 | [29] |
| ISS | 8331-8353 | [51] |
| SD2 G4 | 4968-4971 | [29] |
| **Other^3^** |  | Reviewed in [2, 21] |
| TAR, Poly A, Psi. | 1-811 |  |
| GagPol frameshift | 2085-2136 |  |
| RRE | 7705-8059 |  |
| 3’PPT | 9553-9586 |  |
| cPPT | 4781-4799 |  |
| CTS | 4883-4898 |  |

^1^ Coordinates are given for positions in the HIV-1_NHG_ reporter virus (S3 Data, S4 Data) and reflect sequences that were not changed in the mutant proviruses rather than precisely demarcating the locations of the named elements.

^2^Splice donors were maintained in the synonymously mutated viruses by leaving the 3 and 6 nucleotides positioned 5’ and 3’ to the splice site intact. Splice acceptors were maintained by leaving the PPT, branch point as well as 3 nucleotides and 1 nucleotide positioned 5’ and 3’ to the splice site intact. Cnonical splice sites are indicated. Cryptic splice sites, used at extremely low frequency (D1a, A1a and D2b) [37, 52] were not considered in the mutagenesis strategy.

^3^Splicing regulatory signals not yet reported at the time of mutant design were altered by synonymous mutagenesis
