# Supplementary material for: Global synonymous mutagenesis identifies cis-acting RNA elements that regulate HIV-1 splicing and replication
Source: PLoS Pathog. 2018 Jan 29;14(1):e1006824. doi: 10.1371/journal.ppat.1006824 (PMC5805364; doi:10.1371/journal.ppat.1006824)
Supplement: S2 Table — (DOCX) [file ppat.1006824.s002.docx]

**S2 Table. Positions of sequences targeted in synonymously mutated HIV-1**

| **Primary^1^ Mutant** | **5' end** | **3' end** | **Secondary^2^ mutants** | **5' end** | **3' end** |
| --- | --- | --- | --- | --- | --- |
| **A** | 811 | 1146 | AA | 811 | 972 |
|  |  |  | AC | 811 | 891 |
|  |  |  | AD | 894 | 972 |
|  |  |  | AB | 975 | 1146 |
|  |  |  | AE | 975 | 1056 |
|  |  |  | AF | 1060 | 1146 |
| **B** | 1149 | 1506 | BA | 1149 | 1326 |
|  |  |  | BC | 1149 | 1221 |
|  |  |  | BD | 1227 | 1326 |
|  |  |  | BB | 1329 | 1506 |
|  |  |  | BE | 1329 | 1392 |
|  |  |  | BF | 1398 | 1506 |
| **C** | 1515 | 2082 |  |  |  |
| **D** | 2294 | 2837 |  |  |  |
| **E** | 2846 | 3344 |  |  |  |
| **F** | 3350 | 3839 |  |  |  |
| **G** | 3848 | 4343 |  |  |  |
| **H** | 4349 | 4844 |  |  |  |
| **I** | 4847 | 5345 | IA | 4847 | 5039 |
|  |  |  | IC | 4847 | 4940 |
|  |  |  | ID | 4943 | 5039 |
|  |  |  | ID1 | 4943 | 4991 |
|  |  |  | ID2 | 4992 | 5039 |
|  |  |  | IB | 5097 | 5343 |
|  |  |  | IE | 5097 | 5220 |
|  |  |  | IF | 5223 | 5343 |
|  |  |  | IF1 | 5223 | 5274 |
|  |  |  | IF2 | 5280 | 5343 |
| **J** | 5352 | 5783 | JA | 5352 | 5557 |
|  |  |  | JC | 5352 | 5454 |
|  |  |  | JC1 | 5352 | 5376 |
|  |  |  | JC2 | 5394 | 5413 |
|  |  |  | JC2A | 5394 | 5403 |
|  |  |  | JC2B | 5406 | 5413 |
|  |  |  | JC3 | 5439 | 5454 |
|  |  |  | JD | 5457 | 5558 |
|  |  |  | JD1 | 5457 | 5502 |
|  |  |  | JD2 | 5508 | 5557 |
|  |  |  | JB | 5621 | 5783 |
|  |  |  | JE | 5621 | 5699 |
|  |  |  | JF | 5705 | 5783 |
| **K** | 5786 | 6219 | KA | 5786 | 5898 |
|  |  |  | KC | 5786 | 5827 |
|  |  |  | KD | 5856 | 5898 |
|  |  |  | KD1 | 5856 | 5875 |
|  |  |  | KD2 | 5876 | 5898 |
|  |  |  | KD2A | 5876 | 5883 |
|  |  |  | KD2B | 5889 | 5898 |
|  |  |  | KB | 6069 | 6219 |
| **L** | 6307 | 6805 |  |  |  |
| **M** | 6808 | 7306 |  |  |  |
| **N** | 7312 | 7702 |  |  |  |
| **O** | 8065 | 8329 |  |  |  |
| **P** | 8650 | 8790 |  |  |  |

^1^Primary mutants correspond to the mutant blocks (A though P) depicted in Figure 1A, the nucleotide coordinates of the 5’ end and 3’ end of the mutated segments in the each of the HIV-1_NHG_ –derived proviral plasmids (see S3 Data, S4 Fig) is given.

^2^Secondary mutants refer to the subdivided mutant blocks depicted in Figures 5, 6, 7 and 8. Again the positions in the 5’ end and 3’ end of the mutated segments in the each of the HIV-1_NHG_ –derived proviral plasmids (see S3 Data, S4 Fig) is given.
