# Supplementary material for: Global synonymous mutagenesis identifies cis-acting RNA elements that regulate HIV-1 splicing and replication
Source: PLoS Pathog. 2018 Jan 29;14(1):e1006824. doi: 10.1371/journal.ppat.1006824 (PMC5805364; doi:10.1371/journal.ppat.1006824)
Supplement: S3 Table — (DOCX) [file ppat.1006824.s003.docx]

**S3 Table. MaxEnt scores for canonical and cryptic splice acceptors and donors in wild type and mutated sequence.**

| **Donor** | **MaxEnt Score^1^** | **Sequence** |
| --- | --- | --- |
| **D1** | **10.1** | **CTGGTGAGT** |
| *D1169 (wt)* | *7.16* | *CAGGTCAGC* |
| *D1509 (wt)* | *-4.77* | *CTAGTACCC* |
| *D1725 (wt)* | *7.3* | *GAGGTAAAA* |
| *D1a (wt)* | *10.77* | *CAGGTAAGA* |
| *D1a (mut)* | *5.28* | *CAGGTTCGC* |
| **D2 (wt)** | **5.73** | **AAGGTGAAG** |
| *D2b (wt)* | *5.99* | *CAGGTGATG* |
| **D3 (wt)** | **9.45** | **AAGGTAGGA** |
| **D3 (G5463A mut)** | **3.63** | **AAAGTAGGA** |
| **D4 (wt)** | **9.07** | **GCAGTAAGT** |
| **Acceptor** | **MaxEnt** | **Sequence** |
| *A955 (wt)* | *-9.88* | *CCTTTTAGAGACATCAGAAGGCT* |
| *A955 (mut)* | *1.75* | *GCTACTTGAAACTTCTGAAGGAT* |
| *A1231(wt)* | *-2.94* | *GTTTTCAGCATTATCAGAAGGAG* |
| *A1231 (mut)* | *7.03* | *GTTCTCTGCTCTTTCTGAAGGCG* |
| *A1231 (mut, T1311C)* | *6.09* | *GTTCTCTGCCCTTTCTGAAGGCG* |
| *A1a (wt)* | *2.47* | *ATACTTCCTCTTAAAATTAGCAG* |
| *A1a_mut* | *-3.69* | *TTATTTTCTACTTAAACTTGCTG* |
| **A1 (wt, IB mut)** | **6.41** | **AATTTTCGGGTTTATTACAGGGA** |
| **A1 (I, IA mut)** | **7.41** | **AATTTTCGCGTTTATTACAGGGA** |
| **A1 (IA mut, T4904A)** | **6.57** | **AATTTTCGCGTATATTACAGGGA** |
| **A1 (IB mut, G4912A)** | **-2.34** | **AATTTTCGGGTTTATTACAAGGA** |
| **A2 (wt)** | **9.43** | **CTATTTTGATTGTTTTTCAGAAT** |
| **A2 (J mut)** | **9.71** | **TTACTTCGATTGTTTTTCAGAAT** |
| **A3 (wt, K mut)** | **9.76** | **CTGCTGTTTATCCATTTCAGAAT** |
| **A3 (K mut, C5774T)** | **10.05** | **CTGCTGTTTATCCATTTTAGAAT** |
| **A5 (wt)** | **4.01** | **TTAGGCATCTCCTATGGCAGGAA** |
| **A7 (wt)** | **7.15** | **ATTCACCATTATCGTTTCAGACC** |
