# Supplementary figures and images for: Global synonymous mutagenesis identifies cis-acting RNA elements that regulate HIV-1 splicing and replication
Source: PLoS Pathog. 2018 Jan 29;14(1):e1006824. doi: 10.1371/journal.ppat.1006824 (PMC5805364; doi:10.1371/journal.ppat.1006824)

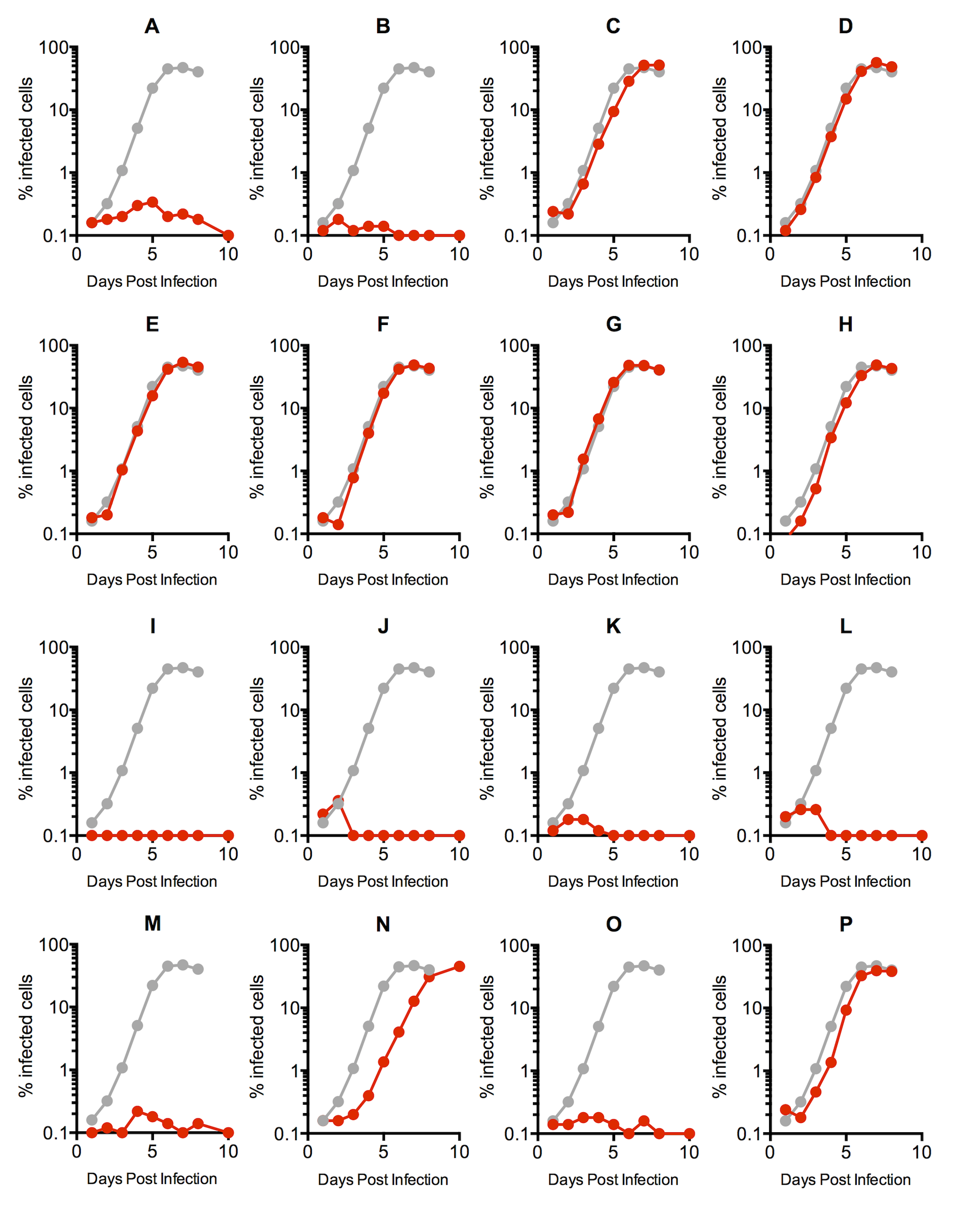

Supplement: S1 Fig — (A-P) MT4 cells were infected with the indicated virus (harvested from the supernatant of 293T cells transfected with each of the WT(HIV-1NHG) mutant (A-P) proviral plasmids at an MOI of 0.002. Aliquots of infected cells were withdrawn each day, fixed in 4% PFA and the proportion of infected cells determined by FACS analysis of GFP expression. (TIFF) [file ppat.1006824.s006.tiff]

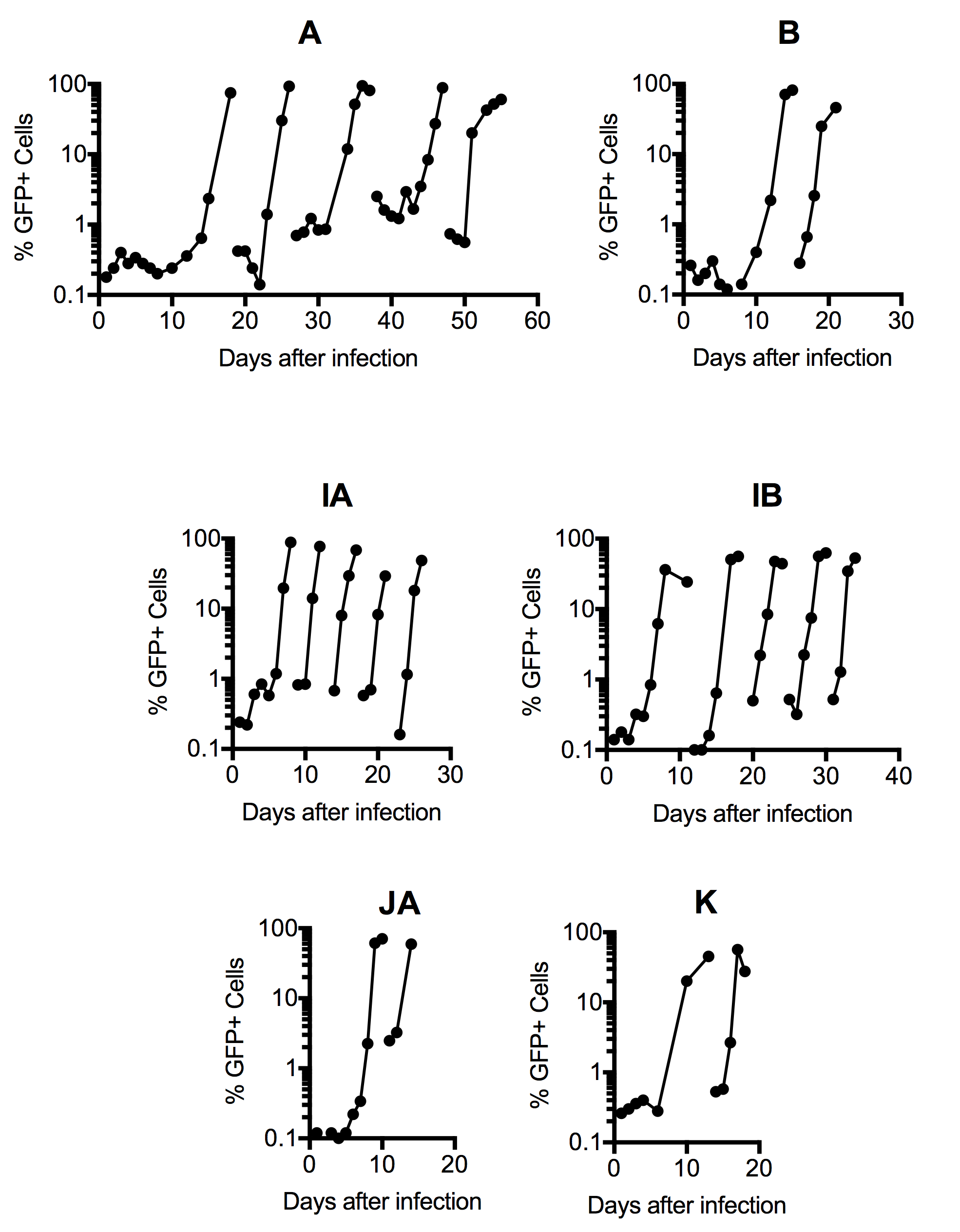

Supplement: S2 Fig — MT4 cells were infected with the mutant viruses (A, B, IA, IB, JA, K, as indicated, harvested from the supernatant of 293T cells transfected with each of the indicated mutant proviral plasmids). Aliquots of infected cells were withdrawn each day, fixed in 4% PFA and the proportion of infected cells determined by FACS analysis of GFP expression. (TIFF) [file ppat.1006824.s007.tiff]
